# Genetic Landscape of Common Epilepsies: Advancing towards Precision in Treatment

**DOI:** 10.3390/ijms21207784

**Published:** 2020-10-21

**Authors:** Sarita Thakran, Debleena Guin, Pooja Singh, Priyanka Singh, Samiksha Kukal, Chitra Rawat, Saroj Yadav, Suman S. Kushwaha, Achal K. Srivastava, Yasha Hasija, Luciano Saso, Srinivasan Ramachandran, Ritushree Kukreti

**Affiliations:** 1 Genomics and Molecular Medicine Unit, Institute of Genomics and Integrative Biology (IGIB), Council of Scientific and Industrial Research (CSIR), Delhi 110007, India; saritathakran93@gmail.com (S.T.); debleena.guin@igib.in (D.G.); poojasinghbhu23@gmail.com (P.S.); priyanka3811053@gmail.com (P.S.); samiksha@igib.in (S.K.); chitra.rawat@igib.in (C.R.); sarojyadav0021@gmail.com (S.Y.); 2 Academy of Scientific and Innovative Research (AcSIR), Ghaziabad 201002, India; ramu@igib.in; 3 Department of Bioinformatics, Delhi Technological University, Shahbad Daulatpur, Main Bawana Road, Delhi 110042, India; yashahasija@dtu.ac.in; 4 Department of Neurology, Institute of Human Behaviour and Allied Sciences, Dilshad Garden, Delhi 110095, India; sumankushwaha@gmail.com; 5 Department of Neurology, All India Institute of Medical Sciences, Ansari Nagar, New Delhi 110029, India; achalsrivastava@hotmail.com; 6 Department of Physiology and Pharmacology “Vittorio Erspamer”, Sapienza University of Rome, P. le Aldo Moro 5, 00185 Rome, Italy; luciano.saso@uniroma1.it; 7 G N Ramachandran Knowledge Centre, Council of Scientific and Industrial Research (CSIR)—Institute of Genomics and Integrative Biology (IGIB), New Delhi 110007, India

**Keywords:** common epilepsies, epilepsy, seizures, genetics, genetic generalized epilepsy, genetic biomarker, prognosis, precision treatment, molecular markers, ion channel receptors

## Abstract

Epilepsy, a neurological disease characterized by recurrent seizures, is highly heterogeneous in nature. Based on the prevalence, epilepsy is classified into two types: common and rare epilepsies. Common epilepsies affecting nearly 95% people with epilepsy, comprise generalized epilepsy which encompass idiopathic generalized epilepsy like childhood absence epilepsy, juvenile myoclonic epilepsy, juvenile absence epilepsy and epilepsy with generalized tonic-clonic seizure on awakening and focal epilepsy like temporal lobe epilepsy and cryptogenic focal epilepsy. In 70% of the epilepsy cases, genetic factors are responsible either as single genetic variant in rare epilepsies or multiple genetic variants acting along with different environmental factors as in common epilepsies. Genetic testing and precision treatment have been developed for a few rare epilepsies and is lacking for common epilepsies due to their complex nature of inheritance. Precision medicine for common epilepsies require a panoramic approach that incorporates polygenic background and other non-genetic factors like microbiome, diet, age at disease onset, optimal time for treatment and other lifestyle factors which influence seizure threshold. This review aims to comprehensively present a state-of-art review of all the genes and their genetic variants that are associated with all common epilepsy subtypes. It also encompasses the basis of these genes in the epileptogenesis. Here, we discussed the current status of the common epilepsy genetics and address the clinical application so far on evidence-based markers in prognosis, diagnosis, and treatment management. In addition, we assessed the diagnostic predictability of a few genetic markers used for disease risk prediction in individuals. A combination of deeper endo-phenotyping including pharmaco-response data, electro-clinical imaging, and other clinical measurements along with genetics may be used to diagnose common epilepsies and this marks a step ahead in precision medicine in common epilepsies management.

## 1. Introduction

Epilepsy, one of the common neurological disease, is characterized by recurrent unprovoked seizures, affecting people of all age, gender, race and geographical location. Nearly 50 million people are affected worldwide with a prevalence rate of 5–10 per 1000 people [1] which accounts for more than 0.5% of the global burden of the disease [2]. Due to the vast heterogeneity associated with the genetics of common epilepsies, it is diagnosed by clinical phenotyping. There are other risk factors like age at onset, and comorbidities (such as psychiatric disorder, intellectual disability and others) that contribute to epilepsy etiology. With time, the disease classification has been dynamic, in coherent with the updated research findings. Based on seizures, epilepsy classification has evolved into different types which are: focal epilepsy, generalized epilepsy, combined focal and generalized epilepsy, and unknown epilepsy. Additionally, according to etiology it is classified as structural, genetic, infectious, metabolic, immune and unknown [3]. 

**Figure 1:**
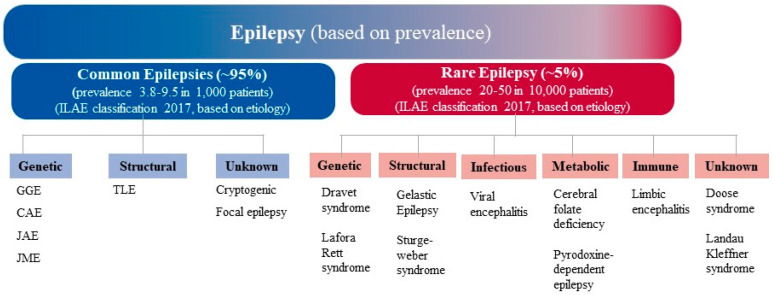
Classification of epilepsy based on its prevalence. Classification of epilepsy based on prevalence of the disease in the global population, into common and rare epilepsies. Common epilepsy is more prevalent in people with epilepsy (~95%) and around ~5% people with epilepsy suffer from rare epilepsy syndromes. According to the latest International League Against Epilepsy (ILAE) classification in 2017, epilepsy is classified based on etiology into genetic, structural, infectious, metabolic, immune and unknown. Different types of are represented under each of these sub-categories. GGE: Genetic generalized epilepsy, CAE: Childhood absence epilepsy, JAE: Juvenile absence epilepsy, JME: Juvenile myoclonic epilepsy, TLE: Temporal lobe epilepsy.

Some of these epilepsy types are highly prevalent in the population than others, and are therefore known as common epilepsies. Such epilepsies are multifactorial and exhibit complex pattern of inheritance, unlike rare epilepsies that display Mendelian inheritance. Of the total patients with active epilepsy (patients who are diagnosed with epilepsy or seizure disorder and either are currently taking medication to control it, or having one or more seizures in the past year, or both), approximately 95% are affected with common epilepsies whereas, 5% suffer from the rare form. The common and rare epilepsies are represented as in Figure 1 as per the latest International League Against Epilepsy (ILAE) etiologic classification of epilepsy, albeit it should be noted that there is no formal classification of rare epilepsy syndromes by ILAE. Common epilepsies broadly comprise generalized and focal epilepsies. Generalized epilepsy encompass genetic generalized epilepsies (GGEs), with its sub-types like juvenile myoclonic epilepsy (JME), childhood absence epilepsy (CAE), juvenile absence epilepsy (JAE), and epilepsy with generalized tonic-clonic seizure (EGTCS) on awakening. Similarly, localization based focal epilepsies include temporal lobe epilepsy (TLE) and cryptogenic focal epilepsy (CFE) [4]. However, of the total GGEs, 1–2% include rare monogenic epilepsies such as autosomal dominant nocturnal frontal lobe epilepsy, benign familial neonatal seizures, early onset epilepsy, myoclonic astatic epilepsy, epilepsy with myoclonic absences, eyelid myoclonic with absences and absence status epilepsy [5,6]. Findings from traditional twin studies and familial aggregation studies revealed that genetic factors can contribute to both focal as well as generalized epilepsies [7]. Clinical studies also suggested that one or more genetic factors are involved in approximately 70–80% of the epilepsy cases, whereas the remaining 20–30% of cases clearly hold an acquired factor such as tumor, stroke, or head injury [8]. Several efforts have been made to identify the genetic variants that are associated with etiology of epilepsy or have an impact in the disease development. Advancement in genomic technologies like sequence-based approaches including massive parallel sequencing, whole-genome sequencing, whole-exome sequencing and targeted gene panel have catalogued numerous potential genetic variations associated with epilepsy. In addition, technologies like comparative genomic hybridization and single nucleotide polymorphism (SNP) genotyping arrays also allowed genome-wide screening of variants in large cohorts in a cost-effective and time-saving manner. These genetic variants cover a large spectrum of variation, from SNPs to the loss or gain of base pairs along with de-novo variants (genetic variants that are first time detected in proband and are absent in parents’ genome) that are detected in some cases. Copy number variations (CNVs) and rare variants of higher effect size have been identified for GGE, CAE and other common epilepsy phenotypes [9]. Genetic variants associated with epilepsy have been found in hundreds of different genes, which may predict disease related phenotypes. The intermediate domain between genotype and phenotype is occupied by an endo-phenotype (heritable trait which may be biochemical, endocrine, electro-physiological, cognitive, or anatomical in nature) [10]. Hence along with genetics, investigating the basis of endo-phenotype could facilitate the layered understanding of subtle features of specific genes in disease physiology. Mounting evidences indicated that quantitative magnetic resonance imaging (QMRI) can detect aberrant fronto-thalamic structure such as loss in thalamic volume and increased mesio-frontal and fronto-basal grey matter concentration in patients with JME, and the white matter microstructural alterations in mesial temporal lobe epilepsy (MTLE) can serve as potential endo-phenotype [9]. Thus, an integrative understanding of endo-phenotype along with genetic risk factors may improve the disease risk predictability. This is ultimately the resolution of precision therapy and the intention of exploring the disease genetics.

Traditional genetic studies like candidate gene study, linkage study, genome-wide association studies (GWAS) are based on screening of the whole genome for the genotype—phenotype association to get insight about genetic architecture and disease susceptibility of complex disease. Several GWAS have been performed to explore the disease genetics in epilepsy. Initially, the findings of GWAS were largely negative due to the small sample size and high genetic heterogeneity in epilepsy [11,12]. Later, several international consortiums joined hands for integrative efforts to explore the disease genetics and its implications. These collaborative efforts of different groups working in the international epilepsy research community made two major advancements in the field. First, large sample size in studies assured a sufficient statistical power for successful genetic association. One of the prominent consortiums established to address the inconsistencies in genetic data is the ILAE. This group published the largest GWAS to date that included 15,212 epilepsy cases and 29,677 controls resulting in the identification of 16 statistically significant risk alleles [13]. Secondly, the consortiums provided a broader phenotypic spectrum by utilizing multi-disciplinary approaches to understand the disease heterogeneity. With this iterative process of cooperative effort, ILAE came up with the newer classifications of epilepsy based on seizure type, disease etiology and clinical factors [14] after almost three decades since the last ILAE classification in 1989 [15]. Several other consortiums have laid the foundation in this field, for example Epi4K majorly explores the genetics of common forms of epilepsy and epileptic encephalopathies, including the prognostic determinants of these disease. Another consortium, Epi25K is the unification of Epi4K, EPIGEN, EuroEPINOMICS, the Epilepsy Phenome/Genome Project, EpiPGX, SANAD, and EpiCURE consortiums which aims to combine the genotype, phenotype, and genomic sequencing data and to perform joint analyses of the data to expedite genetic biomarker discovery in all epilepsies. 

Enormous genetic data is already available on common epilepsies as well as on rare epilepsies across diverse populations which made strides in understanding the genetic architecture of epilepsies. Still much work is needed for identifying the genotype-phenotype correlation for common epilepsies due to the complex nature of the genetics involved. Elucidating the genetic basis of common epilepsy subtypes like rare monogenic epilepsies along with the genome-environment interaction involved in their multifactorial etiology may provide important insights into the pathophysiological mechanism, thus may accelerate the process of accomplishing precision medicine. In this review, we performed a comprehensive state-of-art literature review concerning the genetics of different subtypes of common epilepsies i.e. GGE (CAE, JME, JAE, EGTCS), TLE and CFE. A literature search was performed in PubMed. Further, we used online available databases like ClinVar, Online Mendelian Inheritance in Man (OMIM), Epilepsy Genetic Association Database (epiGAD), Phenotype-Genotype Integrator (PheGenI), DisGeNET and GWAS Catalog to retrieve all genetic markers. Here, we consolidated genetic variants associated with different subgroups of common epilepsies from published evidences. Through this review, we made an attempt to delineate the different subgroups of common epilepsies based on the comprehensive knowledge from genetic association studies. Along with the genes / genetic variants, the substantial role of deep phenotyping may aid in the characterization of the biological functions driving the pathophysiological changes in epilepsy. Identification of such associated genetic variants might presage the end of the diagnostic odyssey and will fuel the field of precision medicine. The discovery of such genetic variants will give direction to unfold the complex genetics of common epilepsies and will encourage researchers to elucidate new diagnostic, prognostic biomarker and new therapeutic targets for epilepsy treatment. This knowledge can be used for clinical practice and genetic counselling. In this paper we have also assessed such genetic markers based on their diagnostic predictability (i.e. sensitivity and specificity) which are commercialized and potentially effective in accurate genetic testing for disease diagnosis by various companies. This will be a leap in epilepsy translation from bench to bedside application.

## 2. Genetic Studies of Common Epilepsies

Epilepsy genetics began its journey from the identification of associated genes and/or their causal variants in Mendelian epilepsies, where a single mutation in a gene can predict the occurrence of disease. However, such strong associations are not detected for the complex forms of epilepsy due to the involvement of multiple factors, including genetic and non-genetic factors. Along with advanced genetic technologies like GWASs and sequencing-based analysis, newer analytical methodologies like polygenic risk score (PRS) can be used. This will help to calculate the genetic load conferred by a set of risk variants to identify the carrier individuals at higher risk. This score can be derived from SNP chips or whole genome sequencing (WGS) for predicting disease risk and estimating heritability. Initial genetic research was driven towards implications of ion channel genes in genetic epilepsies. This led to the emergence of the channelopathy era for genetic epilepsies [16].

**Figure 2:**
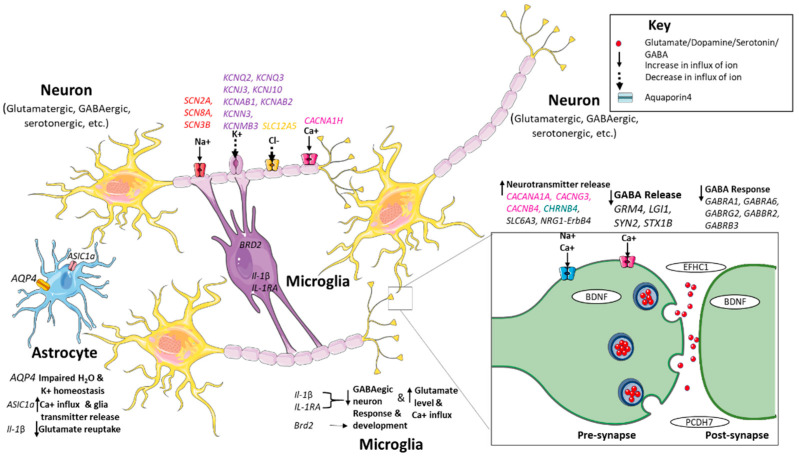
Epilepsy genes: Illustration of several possible patho-genetic mechanisms for common epilepsies. This figure represents the neural network of different cells involved in neurotransmission in brain tissues of epilepsy cases. A broad range of epilepsy mechanisms are implicated due to the identification of genes in the cell body, axon, pre-synapse, post-synapse and in neuroglia. Variation in these genes cause gain or loss of function, culminating in channelopathy disturbance, transporter defects, synaptic dysfunction, DNA repair and chromatin remodeling and transcriptional dysregulation. These defects lead to low threshold action potential and the genes involved in this are *SCN2A*, *SCN8A*, *SCN3B*, *KCNJ3*, *KCNJ10*, *KCNN3*, *KCNMB3*, *CACNA1H*, *AQP4*. Genes such as *CACANA1A*, *CACNG3*, *CACNB4*, *CHRNA4*, *GRM4*, *LGI1*, *ASIC1a*, *STX1B*, *SYN2*, *SLC12A5 ME2*, *ALDH5A*, *Il-1β* and *IL-1RA* and *GABA-A* and *GABA-B* receptor genes affect neurotransmitter synthesis or release either directly or indirectly, which causes an imbalance in excitatory and inhibitory neurotransmitters, causing hyper-excitability in neurons. Other than these, some genes like *PCDH7*, *CPA6*, *EFHC1*, *C3*, *BRD2* and *BDNF* cause defects in synaptic inhibition during brain development, resulting in neuronal hyper-excitability and epilepsy development.

Apart from ion channel genes, which account for a significant proportion of genetic epilepsies, other genes have been identified to be associated with epilepsies with a genetic origin. These genes manifest the diverse mechanisms involved in the pathophysiology and introduced new avenues for therapeutics. Pathways leading to alterations in ion channel structure and their synthesis, in the release and reuptake of neurotransmitters and defects in transporter and post-synaptic receptor activation may result in the loss of function of γ-aminobutyric acid (GABA)-ergic and gain of function of glutamatergic neurotransmission. Such changes may cause an imbalance in the functioning of the excitatory and inhibitory neurons, and eventually disturb the neuronal homeostasis, leading to neuronal hyper-excitability. This is a common patho-genetic mechanism known for all genetic epilepsies [17,18,19] (Figure 2). Genome-wide approaches may assist in the discovery of previously unsuspected markers associated with disease susceptibility, as most genetic variants belong to non-coding regions and cannot justify their biological relevance in disease etiology. This may be due to the small sample size and large heterogeneity associated with epilepsy [11,12]. Genetic findings of all common generalized and focal epilepsies are discussed in detail below to underpin the genetic mechanism of these epilepsies.

### 2.1. Genetic Generalized Epilepsy (GGE)

The most common epilepsy type, GGE, formerly known as idiopathic generalized epilepsy (IGE) covers one fourth of all epilepsies. Absence seizures, myoclonus seizures and generalized tonic-clonic seizures (GTCS) are commonly observed in various combination in GGE patients. Based on seizure types and age at disease onset, GGE is divided into common and rare sub-syndromes [20]. The most common subtypes of GGE are CAE, JME, JAE, and EGTCS [5]. Likewise, the rare sub-syndromes of GGE includes autosomal dominant nocturnal frontal lobe epilepsy, benign familial neonatal seizures and others. Twin studies have shown that epilepsy recurrence rate is higher in monozygotic twin in comparison to dizygotic twins, which provide a strong support for the role of genetics in GGE [6]. In monozygotic twins, the recurrence risk of the common GGE syndromes is 70–95% and that for first degree relatives is 5–8% [21]. Since the risk to have GGE in siblings is lower than the expected rate of a recessive inherited trait (25%) and dominantly inherited trait (50%) [20], this suggests that multiple genes/genetic variants together result in GGE phenotype thereby indicating its polygenic inheritance. Therefore, all susceptible genes can collectively determine the disease risk to GGE [6]. Like other complex diseases, common genetic variants associated with GGE show low impact and rare variants show high impact in epilepsy risk [20]. Based on the GGE subtype, the role of these associated genes in the pathophysiology are discussed in details in the following sections and are summarized in Table 1.

#### 2.1.1. Childhood Absence Epilepsy (CAE)

One of the GGE subtypes, CAE, is idiopathic and characterized by multiple typical absence seizure accompanied by asynchronous, bilateral, 2.5–4 Hz generalized spike and wave epileptiform discharges on the electroencephalogram (EEG). The slow epileptiform episodes are brief discharges (4–20 s) with a frequency of 10–100/day with abrupt onset and termination. This type of epilepsy typically begins between 3-8 years of age with a peak incidence at 5–7 years of age [22] and a prevalence rate of 1–4/50 people with epilepsy (2–8% of total people with epilepsy) [23]. Most of the genes associated with CAE are ion channel genes, like calcium channel, GABA receptor, acetylcholine receptor and so on. The calcium channel genes *CACNA1H* and *CACNG3* are highly associated with CAE [24] particularly in Han Chinese population [25]. Some of the most important genes related known with functional role in CAE are discussed in detail in the following sections. Genetic investigations in patients with CAE have demonstrated the role of GABA A and B receptor genes such as *GABRG2*, *GABRA1*, *GABRB3*, *GABAB1*, *GABAB2* genes which have been implicated in epileptogenesis in such patients. Furthermore, linkage and mutational analysis suggested the involvement of chloride channels genes, *CLCN2*, as a susceptibility locus in a subset of CAE [26].

##### Voltage-Gated Ion Channels

The flow of ions across the neuronal membrane determines the extent of neuronal excitability. Any disturbance in the ion channel structure and function leads to nerve cell hyper-excitability causing epilepsy. Ion channel genes contribute approximately 25% of all the genes identified till date in epilepsy [27]. Based on the nature of ion transport, these genes are divided into voltage-gated (e.g., *CACNA1H, CACNG3, CLCN2*), and ligand-gated (e.g. *CHRNA4*, *GABRA1, GABRB3, GABRG2, GRM4*). 

##### Calcium Channel Genes

Among the voltage-gated ion-channels, CAE is predominantly associated with calcium channels. These channels regulate the release of the excitatory neurotransmitter, glutamate by the pre-synaptic neuron modulating electrogenic properties of dendrites, leading to hyper-excitability [28]. Depending on the voltage required for their activation, calcium channels are divided into low-voltage activated (T-type) channel that belong to CaV_3_ family and high-voltage activated (L-type, P/Q-type, N-type, R-type) channel belonging to CaV_1_, and CaV_2_ family. T-type calcium channels involves *CACNA1G* (CaV_3.1_), *CACNA1H* (CaV_3.2_) and *CACNA1I* (CaV_3.3_) genes encoding the alpha subunit of these channels [29]. A mutational analysis in Han Chinese population of CAE patients identified the presence of 12 missense mutations in *CACNA1H* gene [30]. Another study identified the variant rs2745150 located on intron 11 of *CACNA1H* gene to be significantly associated with CAE [31]. The common variant, rs9934839 in exon 9 and a common haplotype block in *CACNA1H* gene were also significantly associated with CAE in a case-control study in Chinese patients [25]. *In vitro* studies suggested that mutations in the *CACNA1H* gene cause the generation of the slow wave discharges in EEG pattern in absence seizures via increasing its T-type calcium channel activity [32]. In human studies, voltage-gated calcium channel auxiliary subunit gamma 3 (*CACNG3*) gene is found to be associated with CAE, encodes type I transmembrane AMPA (α-amino-3-hydroxy-5-methylisoxazole-4-propionic acid) receptor regulatory protein (TARP). TARP protein regulates both trafficking and channel gating of the AMPA receptors. These AMPA receptor are activated by glutamate binding and have an important role in ictogenesis and epileptogenesis [33,34,35]. These are predominantly present at excitatory synapse. Everett et al. found the significant association of *CACNG3* polymorphism like rs4787924, rs965830, rs2214437 and a haplotype with CAE [26]. All these variants are present in intronic region. These variants might affect the splicing site which may affect TARP protein composition and synthesis ultimately resulting in disturbance in trafficking and gating of AMPA receptor. It may increase AMPA-mediated postsynaptic conductance causing hyper-excitability which generate seizures. Based on these evidences, association of the calcium channel encoding gene variants with human absence epilepsy, specifically CAE was proposed. 

##### Ligand-Gated Channels

**GABA A Receptor Genes:** Another type of ion-channel genes that were observed to be associated with CAE pathophysiology are the ligand-gated GABA receptors (GABARs). The gamma-aminobutyric acid type A receptor subunit alpha1 (*GABRA1*) gene encodes alpha-1 (α1) subunit, of the GABA_A_R protein. GABA_A_R is pentameric having five subunits that arise from seven subunit families: alpha, beta, gamma, delta, rho, theta and epsilon [36]. This receptor acts as a channel for chloride ions to cross the cell membrane. These chloride ions helps in hyper-polarization of post-synaptic membrane potential and inhibits action potential generation [37]. These are chloride channels which act as neurotransmission inhibitor at the synapse [38]. A missense variation R43Q (rs121909673) in *GABRG2* gene was the first GABA type A receptor (GABA_A_R) found to be associated with CAE. *In vitro* studies suggest that the genetic variant may abolish the benzodiazepine (drug which increases GABA activity) sensitivity [39] through imprecise assembly of *GABRG2* subunit with the receptor complex that expedite deactivation of GABAR [38] and also reduces the expression of cell surface GABARs [40,41,42,43]. This termination of the benzodiazepine-induced potentiation of GABARs and deficit of cell surface expression of GABARs may lead to the reduction in synaptic inhibition and neuronal hyper-excitability enhancing risk for CAE [39]. Another study revealed an association between GABA_A_R subunit β3 (*GABRB3*) and CAE [44]. Urak et al. defined 4-haplotypes using 13 SNPs between the exon 1a promoter and the beginning of intron 3 within the *GABRB3* gene region, of which one haplotype was found to be significantly associated with CAE. *In vitro* studies found that this haplotype reduced the expression of *GABRB3*, and could be a potential factor in the development of CAE [45]. Another study suggested that SNPs P11S (rs25409), S15F (rs121913126), and G32R (rs71651682) in the same gene result in hyperglycosylation of the GABRB3 protein causing impairment in maturation and trafficking of GABAR from endoplasmic reticulum to cell surface resulting in reduced GABA-evoked currents leading to generation of absence seizures [46,47].

**Glutamate receptor:** The glutamate metabotropic receptor 4 (*GRM4*) encode the group III mGluR4 (metabotropic glutamate receptor type 4) and regulate the release of glutamate and GABA in the thalamo-cortical network. Studies on animal models have shown that perturbation in mGlu4 receptor function has a role in increasing susceptibility for absence seizure through modulation of glutamate and GABA release [48,49,50]. Genetic variants like rs9380405, rs4711374 are found significantly associated with CAE [51]. These intronic variants might affect the *GRM4* expression which lead to imbalance in glutamate and GABA release and thus increase susceptibility for the epilepsy.

**Acetylcholine Receptor Genes:** A silent polymorphism 594C/T (rs121909580) in the cholinergic receptor nicotinic alpha 4 subunit (*CHRNA4*) gene is found with a higher frequency of T allele in epilepsy cases than control subjects [36]. Hence it can be a susceptible allele for epilepsy which might determine seizure threshold and cause neuronal excitability [52]. 

**µ-Opioid Receptor Gene:** Involvement of µ-opioid receptor gene (*OPRM1*) which encode opioid receptor, has been postulated in the pathogenesis of absence epilepsy. The receptor belongs to the family of seven-transmembrane G protein-coupled potassium channel receptors. This receptor is target for endogenous peptide like β-endorphin which act as neuromodulator [53]. An experiment in WAG/Rij rats which are regarded as a genetic model of absence epilepsy showed that administration of µ- receptor agonist D-Ala-N-methyl-Phe4-Gly-olenkephalin (DAMGO) resulted in dose related increase in slow wave discharge while pretreatment with µ- receptor antagonist, β -funaltrexamine (β –FNA) diminished the action of DAMGO suggesting that activation of µ-opioid receptor increase the epileptic activity. Thus, animal studies provide evidence for their role in absence epilepsy [54,55,56]. A variant Asn40Asp (rs1799971) in opioid receptor has been found significantly associated with CAE phenotype [57]. This variant increases the binding affinity of β-endorphin three time more than the wild type allele. This in turn, activates opioid receptor which alters signal transduction by activation of G protein-coupled potassium channels [56]. This enhances the thalamic neuronal excitability and confers susceptibility to idiopathic absence epilepsy (IAE which constitute both CAE and JAE) [57]. However, replication study on Caucasian population did not find any association of this variant with IAE [58]. It may be due to lack of power for IAE.

##### Solute Carrier Transporters

Solute carrier family 6member 3 (*SLC6A3/DAT1*) gene encoding a dopamine transporter, is a member of the sodium- and chloride-dependent neurotransmitter transporter family. In 3′ UTR region of this gene, a 40bp tandem repeat referred to as a variable number tandem repeat (VNTR), is present in 3 to 11 copies. Variation in the number of repeats, as increase in the nine-copy allele, was found associated with 130 patients with IAE compared with 220 ethnically matched control subjects [59]. This study also found association of this polymorphism with a reduced seizure threshold during alcohol withdrawal [60]. An in vivo study found that dopaminergic transporter *SLC6A3* mRNA levels are significantly lower in the brains of seizure-naïve genetically epilepsy-prone rats [61], suggesting that the nine-copy allele of the 40 bp repeat polymorphism in DAT gene modulates neuronal network excitability and contributes to the epileptogenesis of IAE.

##### Unclassified

Leucine rich repeat LGI family member 4 (*LGI4*) present on chromosome 19q13.1 show an autosomal recessive mode of inheritance. Function of this gene is not known in epilepsy but mutation in this gene might affect neuronal cell migration, axon guidance, or synaptogenesis. A study by Gu. W et al. showed that strong genotypic association exists between CAE and the 1914 GC/AT dinucleotide exchange polymorphism in exon 9 of *LGI4*. High frequency of homozygous 1914 GC/GC genotype in CAE patients suggests an autosomal recessive variant causes greater susceptibility [62].

#### 2.1.2. Juvenile Myoclonic Epilepsy (JME)

Since the first clinical and imaging description of JME was given by Janz and Christian, is also known as JME of Janz [63]. JME is characterized by myoclonic jerks (quick jerks of the arms or legs), GTCSs, and sometimes, absence seizures. Its onset typically begins around adolescence (between 12 and 18 years of age) in otherwise healthy children [64]. JME affects 5–10% of all cases of epilepsy which constitutes 18% of all cases of GGE [65]. Heritability and linkage analysis have revealed various genes associated with JME. GABRA1 at 5q34-q35, EF-hand domain containing 1 (EFHC1) at 6p12 and SLC2A1 at 1p35–p31 are loci discovered through linkage studies. GABRA1, EFHC1, CLCN2 are putative gene for JME while CACNB4 is not considered putative gene, because it has not been replicated [66]. rs3743123 (CX36), rs2029461 (GRM4), rs3918149 (BRD2) showed significant association with JME in more than one population [67]. Variation in EFHC1 is most commonly observed in families with JME [68,69]. This gene encodes microtubule-associated protein which is involved in cell division and neuronal migration. In vitro studies showed that EFHC1 variants cause disruption of radial glial scaffold which are progenitor cells for cortical development and thus impair radial migration [70,71]. This causes micro-dysgenesis as observed in JME patients [72]. Therefore, these defects during corticogenesis may damage epileptic circuitry during brain development [73].

##### Potassium Ion Channels

Potassium ion channel is one of the most divergent ion channel family. The KCNQ2 and KCNQ3 (potassium voltage-gated channel subfamily Q member 2 and member 3, respectively) encoding protein subunits KV7.2 and KV7.3 are found expressed throughout different brain regions which can form homo and hetero-tetrameric channels. These channel conduct slowly activating and deactivating current elicited at subthreshold membrane potentials, the so-called M-current. These M-currents are required for the control of membrane potential and prevent neuronal firing. Polymorphisms rs1801545 and rs74582884 in KCNQ2 and KCNQ3 respectively are found to be associated with JME [74]. Other potassium ion channel genes are also found associated with different GGE which are tabulated in Table 1. These variants might affect the channel gating and thus play a role in epilepsy etiology.

**Table 1:** Overview of the common epilepsyassociated genetic variants.

Sl. No.	Gene/Loci	Gene Family	SNP/Genetic Variant	Risk Allele	Protein Change	GMAF	*p*-Value	OR (CI)	Country	Reference
**GGE**										
1	*SLC4A3*	Solute carrier transporter family	2600G/A	A	A867D	-	0.021	1.48 (1.03–2.14)	Germany	[75]
2	*CACNA1A*	Calcium channel	SNP 8 (SNP in exon 8)	A	-	-	0.00033	1.8 (1.3–2.4)	USA	[76]
3	*CHRNA4*	Acetylcholine receptor	rs1044396	T	S543S	0.323	0.0126	4.9 (1.71–14.04)	Taiwan	[77]
			rs1044394	T	-	0.136	0.02	3.57 (1.31–9.72)	Germany	[52]
4	*D18S474 locus/18q12*	-	-	D18S474 8- and 9-	-	-	<0.001	-	Italy	[78]
5	*GABRA6*	GABA A receptor	rs3219151	T	-	0.43112	<0.001	3.6 (2.1–5.9)	South India	[79]
6	*GABARG*	GABA A receptor	rs211037	T	-	0.371605	0.004	7.36	Egypt	[80]
7	*GRIK*	Glutamate receptor	GRIK tetra-nucleotide polymorphism	9 repeat allele	-	-	0.004	1.26 (1.08–1.47)	Germany	[81]
8	*GRM4*	Glutamate receptor	rs9380405	T	-	0.69	0.003	-	Germany	[51]
			rs937039	G	-	0.257987	0.0038	-		
			rs2451334	A	-	0.163339	0.0118	-		
			rs2029461-rs2451334-rs745501-rs2499697-rs937039	TGTAA	-	0.402157,0.163339,0.330471, 0.078474, 0.25798	0.0069	3.54 (1.42–8.83)	Jordan	[82]
9	*Haptoglobin (Hp)*	-	Hp*1	Hp*1	-	-	<0.001 (Hp*1/*1 vs. other types of epilepsy vs. controls in individuals containing *B/*B genotype in ACP1	0.72 (0.011–0.58)	Italy	[83]
10	*KCNJ3*	Potassium channel gene	T1038C	T	-	-	0.051	1.4 (1.0–1.9)	UK	[84]
11	*KCNJ10*	Potassium channel gene	rs1130183	C	R271C	0.014776	0.03	0.69 (0.50–0.95)	Germany	[85]
12	*KCNMB3*	Potassium channel gene	delA750	-	-	-	0.016	1.52 (1.05–2.21)	Germany	[86]
13	*KCNQ2*	Potassium channel gene	rs1801545	C		0.069286	0.01	1.62 (1.12–2.34)	Germany	[74]
14	*KCNQ3*	Potassium channel gene	rs74582884	A	P574S	0.0015	0.008	-	India	
15	*ME2*	Enzyme	rs674351- rs584087 - rs585344 - rs608781 - rs642698 - rs674210 - rs645088 - rs649224 - rs654136	A-A-C-A-G-A-C-C-A	-	0.287141, 0.269169, 0.270168, 0.164736, 0.260783, 0.270767, 0.272364, 0.15615	-	6.1 (2.9–12.7)	New York	[87]
16	*MTHFR*	Methylene tetrahydrofolate reductase	rs1801133/677C>T	T	A222V	0.24	0.01	2.26 (1.13–4.5)	Scotland	[88]
17	*COPZ2*	Coatomer subunit zeta-2	rs72823592	A	-	0.103834	9.3 × 10^–9^	0.77 (0.71–0.83)	Austria, Belgium, Denmark, Germany and the Netherlands	[89]
18	*SCN1A*	Sodium channel gene	rs8191987	G	-	0.225439	0.03	-	UK	[90]
			rs16851381	G	-	0.166134	0.05	-		
			rs2298771	G	-	0.2115	0.002	-		
19	*SCN2A*	Sodium channel gene	rs2060199	A	-	0.53	0.04	-		
			rs935403	-	-	-	0.03	-		
			rs3943809	G	-	0.202	0.04	-		
20	*SCN8A*	Sodium channel gene	rs303777	A	-	0.297724	0.007	-		
21	*SLC12A5*	Transporter	c.3145 C > T	T	R1049C	-	0.044	9.61 (0.8–503.6)	Canada	[91]
22	*SYN II*	Membrane trafficking	rs37733634	-	-	-	<0.001	2.57 (2.0–3.2)	South India	[79]
			rs3773364	G	-	0.269968	0.02	1.55 (1.06–2.26)	North India	[87]
23	*TAP-1A*	Transporter	333Val-637Asp	-	333V-637D	-	0.02	0.47	Tunisia	[92]
24	*TAP-1C*	Transporter	333Ile-637Asp	-	333I-637D	-	0.03	4.3		
25	*VAMP2*	Vesicle-associated membrane protein (VAMP)/synaptobrevin family	26 bp Ins/Del	ins/del*	-	-	0.042	0.474 (0.230–0.978)	Turkey	[93]
26	*SYT11*	*Synaptotagmin XI*	33-bp repeats in promoter region	C	-	-	<0.001	2.317 (1.503–3.573)		
27	*VRK2*	Vesicle-associated membrane protein (VAMP)/synaptobrevin family	rs13026414	T	-	0.224641	2.5 × 10^–8^	-	Finland, USA, Australia, Canada, Belgium, UK, Republic of Ireland, China, Hong Kong, USA, Canada	[94]
28	*-*	-	rs2947349	C	-	0.28	1 x 10^–8^	1.23 (1.16–1.31)		
29	*MMP8*	Proteinase	rs1939012	T	-	0.385184	2 x 10^–8^	1.02 (1.07–1.17)		
30	*PCDH7*	Cell adhesion molecule	rs1044352	G	-	0.473243	2 x 10^–7^	1.14 (1.08–1.22)		
31	*-*	-	rs55670112	C	-	0.474441	6 x 10^–8^	0.47 (1.10–1.26)		
**CAE**										
32	*CACNA1H*	Calcium channel gene	rs9934839	G	R603R	0.396565	<0.0001	-	China	[31]
			rs2745150	T	-	0.090256	<0.0001	-		
			rs8044363	C	-	0.332668	0.0242	-		[25]
			rs8043905	A	-	0.330871	0.015	-		
			rs9934839	G	R603R	0.3966	0.012	3.367 (1.307–8.671)		
			rs3751664	T	-	0.046725	0.025	1.760 (1.074– 2.886)		
			c.937A>G	G	M313V	-	0.01	0.070 (0.008–0.619)		
			rs119454947	A	F161L	0	-	-		[30,32]
			rs119454949	A	V831M	0.00002	-	-		
33	*CACNG3*	Calcium channel gene	rs447292-rs4787924-rs2239341-rs1494550-c-597delT- rs965830-rs2214437-rs2238500 and rs4787924, rs965830, rs2214437	A, G, A, T, T, G, A	-	0.348442, 0.46865, 0.277356, 0.240815, 0.470847, 0.470647, 0.427316	<0.005	-	UK, France, Germany, Austria, the Netherlands, Denmark, Sweden, Finland and Italy	[26]
34	*CHRNA4*	Acetylcholine receptor	CfoI bp595	T	-	-	0.0397	3.57 (1.16–11.02)	Germany	[52]
35	*GRM4*	Glutamate receptor	rs2499697	C	-	0.92	0.0021	2.36 (1.34–4.15)	Germany	[51]
			rs2451357	G	-	0.87	0.0466	-		
36	*GABRA1*	GABA A receptor	rs1581220270	c.975del	-	-	-	-	Germany	[95]
37	*GABRB3*	GABA A receptor	rs25409	T	P11S	0.002	-	-	Mexico	[46]
38	*GABRG2*	GABA A receptor	rs1561645243	G	-	-	-	-	Germany	[96]
39	*JRK/JH8*	Nucleic acid binding	Thr456Met	456Met	T456M	-	-	-	France, Switzerland, Italy and the UK	[97]
40	*LGI4*	Leucine-rich repeat LGI	c.1914GC-AT	GC/GC	-	-	0.024	2.57 (1.24–5.33)	Germany, Belgium, Turkey	[62]
41	*OPRM1*	Opioid receptor	rs1799971	G	N40D	0.2234	0.019	2.03 (1.12–3.68)	Germany	[57]
42	*SCL6A3/DAT1*	Transporter	40 base pair VNTR polymorphism	Nine-copy allele	-	-	0.002	2.258 (1.32–3.85)	Germany	[59]
43	*VRK2, ACTG1P22*	Vaccinia-related kinase	rs12185644	C	-	0.35643	5 x 10^–10^	-	Caucasian, Asian and African-American	[13]
44	*ZEB2*	Zfh1	rs13020210	G	-	0.311302	2 x 10^–8^	-		
**JME**										
45	*BRD2*	Nucleic acid binding	rs3918149	A	-	0.161542	0.043	1.93(1.01–3.70)	Ireland	[98]
			rs3918149	A	-	0.161542	0.001	2.63(1.42–4.87)	UK	
			rs3918149	A	-	0.161542	-	2.8(1.19–6.64)	North America	[99]
			rs516535	G	-	0.390775	-	2.05(1.00–4.22)		
			rs635688	T	-	0.390775	-	2.16(1.05–4.42)		
			rs206674	G	-	0.003994	-	2.51(1.20–5.24)		
			rs206787	-	-	0.390575	-	2.21(1.08–4.52)		
			rs3918149	A	-	0.161542	-	2.8(1.19–6.64)		
			rs206777	G	-	0.369409	-	2.29(1.11–4.71)		
			rs497058	T	-	0.389776	-	2.08(1.01–4.28)		
46	*CHRNA4*	Acetylcholine receptor	rs45442394	T	-	0.021166	0.029	1.914 (1.057–3.467)	Poland	[100]
47	*CACNB4*	Calcium channel gene	R482X	T	R482X	<0.006	-	-	United state	[101]
48	*CTF1*	Serum cardiotrophin-1	rs1046276	T	-	0.4113	3 × 10^–11^	-	Europe	[13]
49	*CHRM3*	Cholinergic receptor	rs12059546	G	-	0.3298	4.1 × 10^–8^	1.42	Europe	[89]
50	*CILK1*	Kinase	rs376111440	T	R632	0.000032 (GnomAD)	-	-	United States, Mexico, Honduras, Brazil and Japan	[102]
			rs55932059	A	A615T	0 (ALFA)	-	-		
			rs1554169267	G	K220E		-	-		
			rs765078446	C	K305T	0.000033	-	-		
51	*CX36*	Connexin	rs3743123	TT	S196S	0.308906	0.0195	1.62(1.02–2.57)	Germany	[103]
			rs3743123	TT	S196S	0.308906	0.017	4.3 (1.5–12.3)	UK, Denmark, France, Greece, Portugal and Sweden	[104]
52	*EFHC1*	Signal transduction molecule	rs137852778	T	D234Y	0.00002	-	-	Japan	[105]
			rs137852776	C	F229L	0.0018	-	-		
			rs137852777	A	D210N	0.000058	-	-		
			rs149055334	A	P77T	0.002349	-	-		
			rs79761183	A	R221H	0.009585	-	-		
53	*EFHC2*	Signal transduction molecule	rs2208592)	T	S430Y	0.085298	0.03	2.17 (1.06–4.43)	Germany	[106]
54	*GABRA1*	GABA A receptor	rs121434579	-	A322D	-	-	-	Canada	[107]
55	*GRM4*	Glutamate receptor	rs9380405	T	-	0.310304	0.0106	1.33 (1.07–1.65)	Germany	[51]
			rs4711374	T	-	0.301717	0.0266	-		
			rs1466650	T	-	0.404153	0.042	-		
			rs11753413	T	-	-	0.0294	-		
			rs2029461	G	-	0.402157	0.0204	-		
			rs2029461	AG	-	0.402157	0.005	1.641 (1.238–2.175)	India	[108]
			rs2029461-rs2451334-rs745501-rs2499697-rs937039	ACAAA	-	0.402157, 0.163339, 0.330471, 0.0784747 0.257987	<0.0001	0.4907 (0.3475–0.6927)		
				ACACA	-		0.00047	2.5490 (1.5119–4.2973)		
				ATAAG	-		<0.0001	4.8533 (2.2672–10.3895)		
				GCACA	-		0.004525	0.4125 (0.227–0.7495)		
56	*KCNJ10*	Potassium channel gene	rs1130183	T	R271C	0.014776	0.011	0.59 (0.37–0.95)	Germany	[85]
57	*KCNQ2*	Potassium channel gene	rs1801545	C	-	0.069286	0.022	-	Germany	[74]
58	*hSKCa3*	Potassium channel gene	polyglutamine CAG tract	CAG_16_	-	-	0.018	1.198	South India	[77]
				CAG_18_	-	-	0.019	1.178		
				CAG_19_	-	-	<0.00001	0.514		
59	*HLA*	HLA complex	HLA-DRB1*1301-1302	DRB1* 1301-1302	-	-	<0.017	6.6	USA	[109]
			HLA-DQB1*0603-0604	DQB1* 0603-0604	-	-	<0.005	13.8		
			HLA-DQ1	DQ1	-	-	<0.01	-	Japan	[110]
			HLA-DQ3	DQ3	-	-	<0.02	-		
			HLA_DRW13	DRW13	-	-	0.002	4.85 (1.70–14.0)	Saudi Arabia	[111]
60	*SLC2A1*	Transporter	rs387907313	T	R232C	0.000008	-	-	Italy	[93]
61	*TAP-1*	Transporter	333Val-637Asp	-	333V-637D	-	0.007	2.61 (1.27–5.33)	France	[112]
		Transporter	333Ile-637Gly	-	333I-637G	-	0.02	2.30 (1.11–4.77)		
		Transporter	333Val-637Asp	-	333V-637D	-	0.04	0.4	Tunisia	[92]
		Transporter	333Ile-637Asp	-	333I-637D	-	0.03	6.36		
62	*STX1B*	Membrane trafficking	rs1046276	T	-	0.411342	3 × 10^–11^	-	Finland, USA, Australia, Canada, Belgium, UK, Republic of Ireland, China, Hong Kong, Canada	[13]
**JAE**										
63	*INHA*	-	rs7588807	G	-	0.4722	-	-	Turkey	[113]
**TLE**										
64	*APOE*	Apolipoproteins	ApoE-epsilon-4	epsilon4	-	-	0.004	-	Australia	[114]
			ApoE-epsilon-4	epsilon4	-	-	>0.05	1.06 (0.38–2.95)	Turkish	[115]
65	*ASC1a*	Sodium channel	rs844347	A	-	0.224641	0.004	1.516 (1.142–2.013)	China	[116]
			rs844347	A	-	0.224641	0.002	1.628 (1.193–2.222)		
66	*ALDH5A1*	Enzyme	rs1883415	C	-	0.289537	0.0019	-	Germany	[117]
67	*AQP4*	Water channel	ss119336753, ss119336754 and rs1058424	-	-	0.228834	<0.05	-	Norway	[118]
68	*BDNF*	Nucleic acid binding	rs6265	A	V66M (M66 protective)	0.201278	0.012	1.21 (1.04–1.41)	China	[119]
			rs6265	A	V66M (M66 protective)	0.201278	0.636	0.636	Brazil (Caucasian, African, African descent, Asian)	[120]
			rs6265	A	V66M (M66 protective)	0.201278	0.355	-	Europe	[121]
			C240T	T	S80I	-	0.022	-	Japan	[13]
69	*CALHM1*	Calcium channel	rs2986017	A	-	0.128	0.072	1.37 (0.96–1.96)	China	[122]
			rs11191692	A	-	0.298522	0.003	1.35 (1.103–1.653)	China	[123]
			rs11191692–rs729211–rs2986016–rs2986017	G, G, G, T	-	0.298522, 0.363618, 0.119808, 0.127995	0.0029	2.09 (1.27–3.42)		
			rs11191692–rs729211–rs2986016–rs2986017	G, A, G, C	-	0.298522, 0.363618, 0.119808, 0.127995	0.0106	0.7 (0.53–0.92)		
70	*C3*	Immune	Dinucleotide (CA) repeat	-	-	-	<0.05	-	Spain	[124]
71	*CPA6*	Enzyme	rs114402678	T	A270V	0.00359	-	-	Morocco	[125]
			rs61738009	A	G267R	0.001398	-	-		
72	*GABBR1*	GABA A receptor	G1465A	A/G	-	-	<000.1	-	Italy	[126]
			G1465A (with drug-resistant TLE)	A	-	-	0.003	6.47 (2.02–20.76)		
			G1465A	A/G	-	-	<0.0001	10.01 (3.98–25.18)	Argentina	[127]
73	*GABRB3*	GABA A receptor	rs4906902	-	-	0.199681	0.5498		Germany	[117]
74	*GABBR2*	GABA A receptor	rs967932	A	-	0.157149	0.018	1.305 (1.048–1.624)	China	[128]
75	*GAL*	Galanin	C116A	-	A39E	-	-	-	Geneva	[129]
76	*5-HTR2A*	Serotonin receptor	rs6314	T	-	0.074681	0.006	-	Italy	[130]
77	*5-HTTVNTR*	Serotonin receptor	10-repeat allele	-	-	-	0.0187	1.55 (1.07–2.26)	China	[131]
78	*5-HT1A*	Serotonin receptor	rs6295	C	-	0.45	0.048	2.77 (1.01–7.63)	Brazil	[132]
79	*5HT-1B*	Serotonin receptor	rs6296/G861C	G	-	0.33	0.0385	1.574 (1.031–2.402)	Croatia	[129]
80	*5-HTTVNTR*	Serotonin receptor	-	-	-	-	0.0145	RR= 0.21 (0.07–0.65)	Croatia	[133]
81	*IL-1α*	Signaling molecule	rs1800587/IL-1α–889 allele	A	-	0.278	0.018	-	Europe (Caucasian)	[134]
82	*KEAP1*	Kelch-like ECH-associated protein 1	rs1048290	G	-	0.491813	0.04	0.41 (0.20–0.84)	China	[135]
83	*KCNAB1*	Potassium channel gene	rs2280032	A	-	0.479233	0.028	1.84 (1.07–3.18)	Italy	[136]
		Potassium channel gene	rs992353	C	-	0.459265	0.0058	2.25 (1.26–4.04)		
84	*KCNAB2*	Potassium channel gene	rs1386956-rs9816126-rs728382-rs4679773-rs9876870-rs3755631-rs2280561-rs1546750-rs17352408-rs2720281-rs429513-rs2280299-rs2280032-rs992353	GGGGCCTGGGGTTG		0.30611, 0.344249, 0.401358, 0.483826, 0.421326, 0.216653, 0.433706, 0.382987, 0.228634, 0.464058, 0.307308, 0.479233, 0.459265	0.028	12.24 (1.32–113.05)	Italy	[136]
85	*KCNJ10*	Potassium channel gene	rs17375748	-	-	0.024	0.025	-	Norway	[118]
			rs1186685	-	-	0.1829	0.009	-		
			rs4656873	-	-	0.1787	0.019	-		
			rs1186679	-	-	0.1759	0.021	-		
			rs1890532	-	-	0.1905	0.041	-		
			rs946420	-	-	0.1771	0.024	-		
			rs2820585	-	-	0.1771	0.02	-		
86	*NTRK2*	Receptor	rs10868235	T	-	0.351837	0.01	1.9 (1.17–3.09)	Brazil	[137]
87	*NRG1*	Signaling molecule	rs35753505	C	-	-	0.026	0.082 (0.082 (0.009–0.746))	China	[138]
88	*NFE2L2*	Basic leucine zipper (bZIP) proteins	rs7557529–rs35652124–rs6706649–rs6721961–rs2886161– rs1806649–rs2001350–rs10183914–rs2706110	A, A, G, C, A, G, A, G, G	-	0.395168, 0.375599, 0.1451, 0.063299, 0.33631,0.105232, 0.128, 0.23, 0.331669	0.03	7.11 (1.53–32.98)	China	[135]
			rs2706110	A	-	0.331669	0.03	1.95 (1.06–3.58)		
89	*PDYN*	Signaling molecule	-	L-allele	-	-	0.005	-	Austria	[139]
			-	L-allele	-	-	0.061	-	Italy	[140]
			-	L- allele	-	-	0.163	1.6 (0.82–3.31)	Europe (Caucasian)	[134]
90	*PRNP*	Prion protein	rs1799990	G	M129V	0.266	0.021	2.527 (1.11–5.75)	Italy	[141]
			Asn171Ser	-	N171S		<0.0001		Brazil	[107]
91	*TLR4*	Receptor	rs4986790	G	-	0.059904	0.512	1.964 (0.176–21.90)	Europe (Caucasian)	[142]
92	*t-PA*	Tissue plasminogen activator	rs2020918	T	-	-	0.006	2.008 (1.223–3.298)	China	[143]
			rs4646972	311 bp deletion	-	-	0.000	2.007 (1.418–2.840)	China	
93	*SCN1A*	Sodium channel gene	rs7587026	A	-	0.212061	4 × 10^–8^	1.24 (1.15–1.34)	Finland, USA, Belgium, UK, Switzerland, Austria, Republic of Ireland, Australia, Italy, the Netherlands, Portugal, Germany	[144]
			rs3812718	T	-	0.493411	0.0001	1.67 (1.28–2.16)	South India	[76]

Table 1 provides the genetic basis of common epilepsies. There is genetic heterogeneity among these epilepsies. In other words, the same phenotype is caused by variants of different genes. For example, CAE is caused by genes encoding the γ2 and α subunits of γ-aminobutyric acid (GABA_A_) receptors *GABRG2, GABRA1*, and calcium voltage-gated channel subunit alpha 1 H *CACNA1H* and other genetic variants of different genes mentioned in table. These observations illustrate the genetic complexity of the inherited epilepsies and may provide valuable new information for reassessing their classification. In this table, we listed the genes/variants significantly associated with common epilepsy subtypes as well as with GGE as whole obtained from the literature. GGE: genetic generalized epilepsy; CAE: Childhood absence epilepsy; JME: juvenile myoclonic epilepsy; JAE: Juvenile absence epilepsy; TLE: Temporal lobe epilepsy.

##### Signal Transduction Molecule

The EF-hand-containing calcium binding protein encoded by EFHC1 gene, which mediate signaling at the synapse in cooperation with a EFHC1-interaction partner, R-type voltage-dependent calcium channels (VDCC) and has apoptotic activity [145]. Loucks CM et al. demonstrated in C. elegans, that EFHC1 functions within specialized non-motile mechano-sensory cilia where it modulate mechanosensitive calcium channels and at dopaminergic synapse which play a role in neurotransmitter release and thus regulates neuronal excitation Thus, it suggests that EFHC1 protein regulate excitation both at the cilium and at the synapse [146]. This gene is involved in various neuronal functions like regulation of cell division, apoptosis, ion channels, neuronal migration, neurite architecture and neurotransmitter release [73,147] Suzuki et al. observed that in vivo disruption of EFHC1 gene in mice leads to myoclonus seizures and increases seizure susceptibility [105]. Genetic variants like P77T (rs149055334) and R221H (rs79761183) lessen the apoptotic activity of the gene leading to an increase in neuronal cell count and precarious calcium homeostasis by partial reversal of EFHC1-induced Ca2+ influx through CaV2.3 [145]. Reports showed increased density and dystopia of neuron in the brains of JME patients [148]. The combined effect of these result in hyper-stimulation of neurons which give rise to seizure development and JME [145,149]. However in CaV2.3 deficit mice models, no seizure phenotype has been described which may be due to undetected seizure sensitivity [150]. 

##### Nucleic Acid Binding

Bromo-domain containing 2 (BRD2) gene encodes nuclear transcriptional regulator, that belongs to bromo-domains and extra-terminal domain family of proteins which bind specifically to acetylated histones H3 and H4 [151]. These are expressed in developing neural tissue [152]. BRD2 deficit mouse model developed neural tube closure defects and alteration in BRD2 expression during neurodevelopment which may result in increased susceptibility to seizures [153]. BRD2 heterozygous mice showed JME-like behavioral trait, sex specific seizure threshold and seizure related anatomical changes in GABAergic system [154], supporting BRD2 involvement in JME. The genetic variant, rs3918149 within the C-phosphate-G dinucleotides (CpG) island 76 of the BRD2 promoter region was revealed to be an epigenetic variant significantly associated with JME in the Caucasian population. Authors discussed that patients with JME show CpG76 hyper-methylation, possibly leading to decrease in BRD2 transcription [67,99,155]. In an animal model study, BRD2-null mutation (BRD2+/−) in mice causes a decrease in GABAergic neurons along the basal ganglia seizure-controlling pathway and GABA-synthesizing enzyme expression (GAD67), increasing seizure susceptibility and seizure development. This in turn might result in decrease in specific GABAergic neuronal population and enhanced seizure susceptibility [99,154,155]. Variation in promoter may disrupt interaction with other proteins involved in controlling particular stages of brain development. Neural cell overgrowth or lack of apoptosis in specific regions of the brain may occur due to abnormalities in development pathway. These abnormalities result in unorganized neuronal connectivity causing hyper-excitability which leads to seizure development, a mechanism of epileptogenesis [99,149]. Though supporting evidence for BRD2 association with JME was found in British and Irish cohort, no such association was seen in Australian, German and Southern Indian population [98]. Non-Caucasian population failed to support BRD2 association with JME [112,156]. All these evidences suggest that BRD2 in JME, is ethnicity specific, showing differential methylation.

##### GABA A Receptor

GABRA1 gene was initially implicated in familial JME [157]. Mutations in the GABAAR such as Q351X (rs121909674), R43Q (rs121909673) in GABRG2, A322D (rs121434579), S326fs328X in GABRA1, and R220H (rs41307846) in GABRD are majorly involved in reduced protein expression of GABAAR [158]. In vitro studies suggest that A322D missense mutation causes α1 protein mis-folding, due to which α1 subunit is rapidly degraded in endoplasmic reticulum through the ubiquitin–proteasome system [159]. This lowers surface expression of mature protein [160], in turn reducing GABA evoked chloride currents, leading to neuronal disinhibition by preventing hyperpolarization of membrane [161]. Studies have shown that R220H variant of GABRD can be a susceptibility allele for JME [162,163]. In contrast, Lenzen et al. found no association between the R220H variant and JME among 562 German patients and 664 controls [164].

So far, ten GWAS studies have been carried out for epilepsy in general, and two studies found SNPs association with JME. First stage GWAS study of EPICURE included 586 patients with JME and 2461 controls of North Western European ancestry and replication stage included 382 European JME patients with 382 ethnically matched controls. After combined association analysis of both the stages, rs12059546 in M3 muscarinic acetylcholine receptor (CHRM3), reached genome-wide significance [89]. Studies suggested CHRM3 gene to play a role in differential cholinergic modulation in distinct hippocampal cell, which may influence synchronization and excitability of thalamo-cortical circuits and thereby seizure susceptibility [165]. However the results were proved inconclusive in a pilocarpine model [166]. Another genome-wide mega-analysis that included 15,212 people with epilepsy and 29,677 controls, found a significant association of rs1046276 (STX1B) at 16p11.2 locus with JME [13]. This gene has a role in release of neurotransmitter by fusion of presynaptic vesicle membrane [167]. Variant in STX1B may result in hyper-excitability of neuron giving rise to epilepsy [168].

#### 2.1.3. Juvenile Absence Epilepsy (JAE) and Epilepsy with Generalized Tonic–Clonic Seizures (EGTCS)

JAE is a GGE syndrome typically begins between 10 and 16 years of age with a peak at 15 years [169] and is predominantly characterized by absence seizures. Patients may experience other seizure types as GTCS, GTCS on awakening, and myoclonic seizures also. Exact etiology of JAE is not known, but studies have shown genetic variations in genes like voltage-gated sodium channels (CACNB4), ligand-gated ion channels (GABRA1, GRIK1) and EFHC1 genes to be involved in JAE [170]. Sander et al. reported an allelic association with GLU R5 kainate receptor gene (GRIK1 polymorphism), which has a role in excitatory neurotransmission [81]. Another association study revealed a strong association of a common intronic SNP (rs7588807) in the inhibin alpha precursor gene (INHA) with JAE [113]. INHA encodes inhibin protein, which inhibits the secretion of follicle-stimulating hormone (FSH), in turn inducing the production of progesterone and estradiol. This SNP is predicted to exert a direct effect by increasing the brain excitability or an indirect effect on absence seizures as increased production of progesterone enhances slow wave discharge through allo-pregnanolone, a positive modulator of GABAAR [171].

EGTCS commences at 10 to 16 years of age in which GTCS that occur mostly within 2 h after awakening from sleep, hence also known as epilepsy with tonic-clonic seizures on awakening. The genetics of EGTCS is complex. No genetic variants are found linked with EGTCS [172]. Some studies have reported JAE and EGTCS as common epilepsies [173] but role of genetics in their etiology is highly elusive. Hence, we have not discussed these epilepsies in detail.

### 2.2. Temporal Lobe Epilepsy (TLE)

TLE is most common form of partial epilepsy which is often associated with brain injury, head stroke, trauma and infection. Hence, it is classified under symptomatic or structural epilepsy. Based on the seizure origin, TLE is subdivided as mesial, lateral and neocortical. Genetic factors along with other factors like injury, infection or lesions in temporal lobe, are also believed to be involved in its etiology. Hence, to understand the role of genetics in TLE, linkage analysis in families and association studies have been carried out [137,174]. Studies have suggested that LGI1 mutations is linked with autosomal dominant lateral temporal lobe epilepsy. These studies showed that family members of affected person are at high risk than members of healthy individuals. 

#### 2.2.1. Sodium Ion Channels

It is one of the most important class of genes associated with various epilepsy phenotypes [175]. The voltage-gated sodium ion channels consist of large α subunits that associate with β subunits to form voltage gated ion channels [176]. There are several alleles of the alpha subunit gene referred to as SCN1A through SCN11A. Based on the sequence, expression profile and kinetics, sodium channels are distinguished. SCN1A variants are found associated with a continuum of epilepsy phenotypes such as intractable childhood epilepsy with generalized tonic-clonic seizures, including simple febrile seizures or familial fever-related epilepsies referred to as generalized epilepsies with febrile seizures and also a risk factor for common epilepsies like TLE and GGE [177]. A meta-analysis revealed genome-wide significant association of an intronic polymorphism i.e. rs7587026 of SCN1A for mesial temporal lobe epilepsy with hippocampal sclerosis with febrile seizures [144]. Another intronic variant rs3812718 is also found significantly associated with mesial temporal lobe epilepsy with hippocampal sclerosis [76]. This polymorphism disrupt the conserved consensus-site sequence and result in weaker 5’ splice site which increase the expression of exon 5N. Product of this transcript variant of SCN1A cause altered electrical signaling [178].

#### 2.2.2. Calcium Homeostasis Modulator 1 (CALHM1)

Calcium channel encoding gene CALHM1 has a role in calcium-dependent neuronal signaling [179]. Calcium ions have a substantial role in epilepsy development [180]. A polymorphism rs2986017 in this gene interferes with calcium homeostasis and increases amyloid β levels [181]. Experimental data have suggested that overexpression of amyloid β in the brain can cause epileptiform activity and can increase intra-neuronal resting Ca2+ concentration [182] and seizure susceptibility [183]. Increased amyloid β levels in the form of senile plaques have been observed in TLE patients [184]. Another polymorphism in the 3’ UTR of same gene rs11191692 was identified as a risk factor for TLE subjects in North China. This SNP might affect Ca2+ mediated release of excitatory neurotransmitter and also modulates amyloid beta level though precise mechanism is yet to be identified [123]. In vitro studies suggested role of Ca2+ ions in TLE [185] and increased amyloid β level causes aberrant neuronal activity resulting in cortical and hippocampal network susceptible for epilepsy [186]. A replication study in TLE patients from South China failed to support previous finding that CALHM1 contribute substantially to MTLE [122]. Failure to replicate previous studies may be due to underpowered sample size and undetected population stratification. Hence, it is possible that initial finding gave a true association with MTLE [186].

#### 2.2.3. γ- Aminobutyric Acid B Receptor 1 (GABBR1)

An essential component of pre- and postsynaptic GABABR, encoded by GABBR1, is abundantly expressed in temporal lobe structures. This ligand-gated GABABR inhibits release of neurotransmitter from presynaptic neurons and mediates late inhibitory postsynaptic potentials [187]. A missense mutation in exon 7 of GABBR1, c.1465G>A (p.Gly489Ser) was found significantly associated with TLE [126]. In another study subjects carrying heterozygous A allele for this polymorphism had 10 fold increase in risk for MTLE with hippocampal sclerosis [127]. This genetic variant is present in N-terminal extracellular domain of the GABABR, which is the site for ligand binding. Hence, this genetic polymorphism may affect the ligand binding properties which may perturb correct functioning of receptor that would lead to inhibition of GABA release from pre-synapse and promote development of seizures. The association of this genetic variant with TLE did not replicate in other studies and remained inconclusive [128,188,189,190,191,192,193]. A positive association was found for the A-allele of rs967932 (GABBR2) with the risk of TLE in the Chinese population. These patients showed frequent occurrence of GABBR2 haplotype (G-C-A-C, rs3780428-rs1999501-rs967932-rs944688, respectively) predisposing them to the early onset of the disease [128]. The role of this haplotype is not clear. 

#### 2.2.4. Aquaporin

The aquaporin 4 (AQP4) gene encode a protein that acts as water selective channel. AQP4 expression in glial cell (astrocyte) has a role in water and ion homeostasis in brain, as water flux through this channel, is coupled with extracellular K+ clearance through inwardly rectifying K+ channel [194]. In vivo studies have shown that deletion of AQP4 perturb the osmolarity by accumulation of K+, which causes membrane depolarization resulting into synchronous discharge from nerve cells and increase seizure susceptibility [195,196]. Interestingly, AQP4 expression is reduced in the kainate model of epilepsy, and this is also confirmed in AQP4 deficit mice in which seizure susceptibility is increased. This reduced expression of AQP4 might impair water delivery to the extracellular space and increase excitability [197,198]. Eid T. et al. also found that perturbed expression pattern of AQP4 and its anchoring complex in MTLE patients, could underlie the deficiency in water and K+ homeostasis [199]. Heuser et al. indicated three non-coding variants in AQP4 in significant association with TLE [118]. These variants might act as regulatory element and decrease the expression pattern of AQP4.

#### 2.2.5. Serotonin Transporter (5-HTT)

Serotonin neurotransmitter has a substantial role in cortical and subcortical excitatory/inhibitory balance. After its release from pre-synapse, its action is terminated by its reuptake via 5-HTT which is key regulator in serotoninergic neurotransmission and has anticonvulsant property [200]. Two functional polymorphisms of 5-HTT, 5-HTTLPR (an insertion/deletion in 5 UTR) and 5-HTTVNTR (a VNTR in intron 2) were suggested to modulate its transcription and play a role in TLE etiology. Deletion of 44 bp in promoter region generates a 14-repeat variant and its insertion generates a 16-repeat allele in the 5-HTTLPR which are known as short (S, low expressing) and long (L, high expressing) alleles respectively. A significant association for lower frequency of 10 repeat allele at 5-HTTVNTR in TLE patient was found but no association was observed for 5-HTTLPR [133]. A study shown that MTLE-HS homozygous carrier of 12 repeat allele of 5-HTTVNTR had higher risk for non-response to medical treatment compared to 10 repeat allele carrier [201]. Combination of the transcriptionally more efficient 5-HTT genotypes, i.e., 5-HTTLPR L/L and VNTR-2 12/12 was found to be associated with poor response of optimal drug therapy [202]. In contrast to these findings, an association was found between transcriptionally less efficient combined genotypes of 5-HTTLPR and 5-HTTVNTR and TLE [203]. Li et al. found a higher frequency of 10-repeat allele at 5-HTTVNTR whereas Chi et al. found association of 12/12 genotype and allele 12 at 5-HTTVNTR in Han Chinese TLE subjects [131,204]. Stefulj et al. did not find any association between 5-HTTLPR or 5-HTTVNTR and TLE but a high frequency of serotonin receptor 5HT-1B allele 861G was found in the TLE patients of the Croatian population [205].

#### 2.2.6. Prodynorphin (PDYN)

Prodynorphin (PDYN) gene encodes the precursor of anticonvulsant dynorphin opioid peptides. A 68bp tandem repeat element containing binding site for AP-1 transcription factor, is present in the core promoter of PDYN gene, and regulate its expression [206]. This polymorphism causes low expression of this gene. AP-1 has a central role in regulation of seizure-related gene expression by affecting transcriptional machinery. One or two repeats of 68bp named as L-allele, is associated with low PDYN expression, resulting in low prodynorphin level which increase susceptibility for epilepsy. Whereas the H-allele, characterized by three or four repeats of the element is associated with high expression of PDYN gene, causing anti-convulsive effect. Two studies found L-allele to increase the risk of TLE in patients with familial history of seizures [139,140]. This result was not replicated in four independent studies of the Caucasian population for TLE [134,188,207,208]. However, a meta-analysis suggested that the L-allele variant of this promoter polymorphism might contribute as a risk factor for familial-TLE suggesting that further studies are required for acquiring discrete results [209].

#### 2.2.7. Acid-Sensing Ion Channel Subunit 1 (ASIC1)

Acid sensing ion channel subunit 1alpha (ASIC1a) gene encodes a member of the acid-sensing ion channel (ASIC) family of proteins widely expressed in the neurons of the peripheral sensory and the central nervous system [210]. In vitro and in vivo studies indicate that ASIC1a get activated by low extracellular pH value in brain followed by high expression of ASIC1a in hippocampal astrocytes of TLE patients and epileptic mice [211,212].This causes a significant increase of intracellular Ca2+ ion level in astrocytes. Accumulation of Ca2+ ions activates astrocytes to release glio-transmitter like glutamate which promote generation and spread of seizures [213]. On the contrary, Ziemann et al. reported that ASIC1a is highly expressed in GABAergic interneurons which is involved in termination of seizure [214]. The seizure generation and termination depends on the excess expression of ASIC1a on active astrocytes or GABAergic neuron, respectively [213]. A genetic association study in the Han Chinese population showed that rs844347 in the intronic region of ASIC1a gene could be a plausible risk factor for TLE [116]. 

#### 2.2.8. Apolipoprotein E (ApoE)

The gene, ApoE encoding a major apoprotein of the chylomicron, has three major isoforms: ApoEε2, ApoEε3 and ApoEε4. Earlier reports on ApoEε4 association with early onset TLE subjects of Italy was not significant [215]. Later the same allele was found positively associated with early onset TLE subjects in the Australian population [114]. Subsequently, five replication studies in different populations were performed [115,134,188,216,217] but one study suggested association with TLE [134]. Another study reported that ApoEε4 allele in intractable TLE affect both verbal and non-verbal memory performance [218] and increases the risk of post-ictal confusion [219]. While Kauffman et al. did not find any association with the same [220]. A study in the Han Chinese population suggest that ApoEε4 allele is involved in development of TLE in patients with prior brain trauma [221] and is a risk factor for non-lesional MTLE [222]. All these evidences suggest that ApoEε4 allele has a role in TLE. ApoEε4 contributed substantially in neuronal degeneration through promoting intracerebral accumulation of β-amyloid [223], which act as a linker between ApoEε4 and TLE [224]. Kodam A et al. also demonstrated increased production or secretion of amyloid beta related peptides from activated astrocytes to cause neurotoxicity suggesting these peptides have a role in TLE pathogenesis [225].

#### 2.2.9. Neurotrophic Receptor Tyrosine Kinase 2 (NTRK2)

The NTRK2 gene encodes a membrane receptor kinase TrkB [226] that undergoes auto-phosphorylation upon binding of neuron survival factor neurotrophin. TrkB plays an essential role in maintaining synaptic plasticity. Reduction in TrkB receptor expression or its inactivation has been demonstrated to impair seizure induction and epileptogenesis in various in vivo studies. Torres CM et al. performed a case-control study to compare the allelic/genotypic frequencies of multiple polymorphisms of TRKB gene between Caucasian patients with TLE and healthy controls. An increasing statistical trend for T/T genotype of rs10868235 was observed in patient group. Further, analyzing clinical or electrographic variables in the patient group revealed that the patients with A/A genotype for rs1443445 had early age at seizure onset. Also, patients in need of polytherapy had a greater frequency of T-allele for rs3780645 than patients on monotherapy [137].

### 2.3. Cryptogenic Focal Epilepsy (CFE)

Epilepsy which do not meet the criteria of idiopathic/ genetic partial epilepsy and also lack underlying genetic, structural or metabolic etiology is known as cryptogenic focal epilepsy. This epilepsy type does not have a clear etiology hence also known as unknown epilepsy and accounts for a significant proportion of all epilepsies [227]. Harkin et al., identified de novo mutation in SCN1A gene in 22% of CFE patient cohort [228]. Another study of SCN1A confirmed the findings of first study having 12.5% of CFE patients with SCN1A variation [229]. Beside this, no other genes have been found associated with CFE. 

## 3. Genetic Burden of Rare and Common Variants in Common Epilepsies

The most important unresolved question in genetics of epilepsy is, if the genes responsible for rare/monogenic epilepsy also contribute to common epilepsies and to what extent. Another concern is, if the rare variants with large effect size and/or common variants with minimal odds ratio contribute to the common epilepsy disease risk [229,230]. To find the burden of ultra-rare (allele frequencies < 0.0005) genetic variation in common epilepsies, WES was performed coordinated by the Epi4K Consortium. Findings of this study suggested that DEP (Dishevelled, Egl-10 and Pleckstrin) domain containing 5 (DEPDC5), leucine-rich glioma inactivated 1 (LGI1), protocadherin 19 (PCDH19), SCN1A, and glutamate ionotropic receptor NMDA type subunit 2A (GRIN2A) are the five genes which occupied the top genome-wide ranks in order of increasing p-value in individuals with familial non-acquired focal epilepsy. Out of these only DEPDC5 gene showed genome-wide significance. But none of the genes were significant for GGE cases. However, potassium voltage-gated channel subfamily Q member 2 (KCNQ2), SCN1A, and GABRG2 are three established genes among top ten genes for common epilepsies [231]. Independent studies confirmed that pathogenic variants in such genes are ultra-rare in general population and are present not more than once in 60,000 individuals in population database like ExAC [232]. Another study by EuroEPINOMICS consortium found enrichment of rare variants with minor allele frequencies <0.005 in GABAAR encoding 19 genes in GGE patients but did not identify genome-wide burden of rare variants in single gene [233]. To yield more significant result the largest whole exome sequencing was performed for 17,606 individuals by EPI25 consortium. This study found that across all three classes of epilepsy like severe developmental and epileptic encephalopathies, GGE and non-acquired focal epilepsy, gene GABRG2 were enriched for missense variants. GABAergic inhibition plays an important role in epilepsy etiology. The findings of this study also suggest a higher genetic burden of ultra-rare variants on GGE than non-acquired focal epilepsy [234]. All these evidences show that rare variants contribute to common epilepsies.

Common variants that contributes to common epilepsies have so far yielded mostly non-reproducible results. Nonetheless, with the inclusion of larger sample cohorts and use of polygenic risk score studies made better attempt in investigating the attributable common variants with higher statistical rigor. A meta-analysis of GWAS identified two genes SCN1A and protocadherin7 (PCDH7) common for all epilepsies i.e. GGE, focal epilepsy and unknown. Another locus 2p16.1 was found significantly associated with all GGEs, but it was uncertain which of the genes in this loci, VRK2 or FANCL gene, play a role in the same [94]. Association of this locus was also strengthened by one GWAS study and another mega analysis [94]. The VRK2 gene encode a protein kinase involved in signal transduction and apoptosis. This locus (2q24.3) was found to be associated with both GGE and focal epilepsy in two different mega analyses [13,94]. Identification of large number of significant risk allele has become possible due to increasing sample size and through large collaborative work [13]. Common risk variants associated with a disease cannot individually quantify risk because these variants generally have a small effect size [235].

However, polygenic risk score (PRS) can be used to estimate the cumulative effect of thousands of variants into a single score, rather than considering single variant effect in disease as in GWAS. The PRS can be used to distinguish patients with epilepsy (based on severity), individuals who are at risk of developing epilepsy and healthy individuals [236]. This approach highlighted that common variants collectively explain 26% phenotypic variation for all epilepies, 27% for focal epilepsy and, approximately 33% for GGE [13,237]. The estimated contribution of common risk variants obtained by this method is greater than any other analytical method supporting the hypothesis that multiple genes contribute to common epilepsies [238]. Leu et al. calculated the PRS from the GWAS for two main subtype of epilepsy: GGE and focal epilepsy and observed that generalized epilepsy have a significantly higher burden of common risk variants than patients with focal epilepsy [236]. It is also observed that there should be homogeneity between the population from which PRS is calculated and the population on which it is applied, otherwise it results into low predictive power of PRS [239].

Extensive research is required to generate new prediction model to exploit the use of PRS by distinct cohorts from different populations with well-characterized epilepsy phenotypes. A combined risk score including PRS along with weighted scoring of non-genetic risk factors will be the most accurate method for implementing it in clinical practice. This will prove a boon for clinicians as well as for patients in better prognosis and precision treatment. Non-genetic risk factors include individual characteristics (diet, sex, life stage, ethnicity, education), the clinical factors (severity, age at onset, treatment gap, available treatments, comorbidities), community context and values (religion, social networks, support, personal preferences, economic strata), internal milieu of an individual (epigenetic, microbiome profile and metabolome), brain infections and environmental factors, which influence the genetic information and epilepsy risk, hence cannot be neglected [240,241]. In the context of epilepsy, a link between gut microbiome and brain through gut-brain axis have also been established through neuro-immuno-endocrine mediators [242]. This combined scoring of genetic and non-genetic factors will make a paradigm shift from risk prediction and clinical management for rare monogenic epilepsies to complex epilepsies and also help in filling the gap of precision medicine for common epilepsies

## 4. Copy Number Variants (CNVs) in Epilepsy

Apart from SNPs, CNVs have been demonstrated as risk or causal genetic markers in GGE including both in JME and CAE. Development of chromosome microarray and SNP genotyping arrays facilitated genome-wide screening for de-novo CNVs in large cohorts [243]. The first CNV identified was 15q13.3 microdeletion in CHRNA7 is linked with intellectual disability and epilepsy [243]. Sharp et al. described this microdeletion recurrence in people with epilepsy or individuals showing abnormal EEG. CHRNA7 coding for synaptic ion channel protein is important for neuronal signal transmission and has been previously shown to be susceptibility factor for JME and benign epilepsy of childhood. In vivo data also shows its involvement in hyper-synchronous EEG phenotype. Study in the subsequent year also found 15q13.3 microdeletion encompassing the CHRNA7 gene in patients with IGE, supporting role of 15q13.3 microdeletion as a prevalent genetic risk factor for epilepsy. Dibbens et al. further confirmed GGE to be the most frequent phenotype associated with the 15q13.3 microdeletion. With the odd ratio of 68, the deletion indicated any pathogenic lesion predisposing to epilepsy with polygenic inheritance and incomplete penetrance in GGE [244,245]. High density SNP array unveiled microdeletions at 15q11.2 and 16p13.11 to be significantly involved in epileptogenesis in patients with GGE [246]. These three microdeletions are represented as “genetic hotspot” as they share a common genetic architecture. 15q13.3, 15q11.2 and 16p13.11 deletions are primarily risk factors for GGE and also found in patients with focal epilepsy (Table 2). A study including 315 patients with epileptic encephalopathy did not find any of these microdeletions highlighting the fact that common epilepsies have a different genetic architecture than epileptic encephalopathies [247]. Rare exonic deletions in neuronal genes as neuronal adhesion molecule of pre-synaptic terminal i.e. neurexin 1 (NRXN1) [248], neuron specific splicing regulator gene, RNA binding fox-1 homolog 1(RBFOX1) [249], and scaffolding protein in the neuronal post synaptic membrane gephyrin (GPHN) [250] disrupt the exons and are also found to increase the risk of GGE. 

## 5. Clinical Implication and Relevance of Genetic Findings

**Figure 3:**
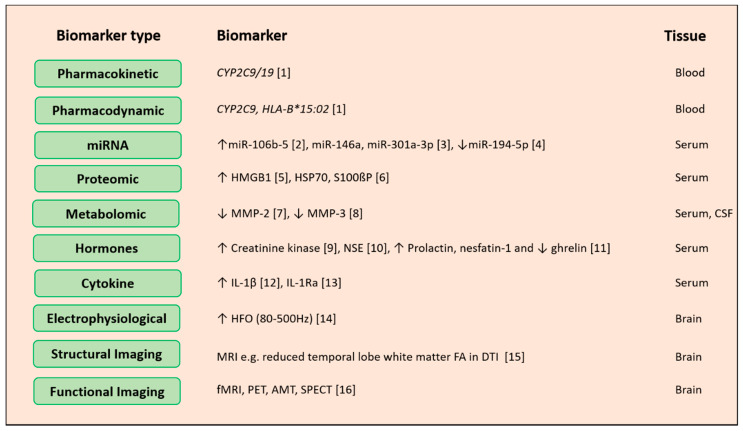
Potential biomarkers for epilepsy diagnosis. Biological levels and sources of epilepsy biomarker omics can be measured across different biological levels around the genome, pharmacogenome, transcriptome, proteome and metabolome. Other than these hormones, cytokines and electrical imaging records can also act as biomarkers for disease prediction, drug response improvement and avoidance of adverse side effects of drugs. Abbreviations: miRNA: microRNA, *CYP2C9:* cytochrome P450 family 2 subfamily C member 9, HLA-B: major histocompatibility complex class I, B, HMGB1: high mobility group box 1, HSP70: heat shock protein 70, S100ßP: S100 calcium-binding protein B, MMP: matrix metallopeptidase 2, NSE: neuron-specific enolase, IL-1β: interleukin-1 beta, IL-1Ra: interleukin-1 receptor antagonist, HFO: high-frequency oscillations, MRI: magnetic resonance imaging, FA: fractional anisotropy, DTI: diffusion tensor imaging, fMRI: functional magnetic resonance imaging, PET: positron emission tomography, SPECT: single photon emission computed tomography.

Remarkable advancements in identifying the causative mutations and technological growth in epilepsy genetics have channelized genetic testing into clinics. Various systemic level biomarker can be used to ensure proper diagnosis of the disease and the prediction of the treatment outcome (prognosis of anti-epileptic drug response) (Figure 3). Genetic biomarker is used to predict the risk occurrence of epilepsy in a person with a family history of epilepsy which is a commonly encountered situation. This can be used for pharmaco-response for selected antiepileptic drugs. Performing testing of specific single gene for diagnostics is no longer a practical approach for complex diseases like common epilepsies. Thus, development of gene panels and beginning of NGS-based or exome sequencing platforms for disease diagnosis has marked its way for a more comprehensive assessment of the disease status. These platforms predict the putative pathogenic variants of genes having a role in specific epilepsy subtype, and also of those genes with no known evidences with disease, but might regulate other genes with known functions in epilepsy. This may help us investigate the potential pathogenicity of such variants. Such studies hint that along with genetic data, clinical phenotyping, family history/ genealogy data together can assist in precision medicine in epilepsy. Clinical phenotyping does not include only neurological examination and tests but also thorough assessment of seizure type, duration and frequency, dysmorphic features, cutaneous signs, congenital malformation, variable symptoms of any other organ or organ system impairment, results of radiological, biochemical and other testing, cognitive functioning information etc. These phenotypes are influenced by several genes, epigenetic and environmental factors. Hence, gene/variant interpretation are crucially dependent on the full phenotypic picture of the patient. Therefore, these genetic variants need to be analyzed and integrated with detailed clinical phenotyping to make a diagnosis. These changes will make improvements in diagnosis and treatment.

Beyond efficient prognosis or diagnosis of the disease, one of the other prime intent behind performing genetic studies is to identify novel evidence-based drug targets for future drug development. It may also allow better designing of clinical trials to standardize drug dosing, treatment outcome evaluation or toxicity profiling with respect to specific phenotypic spectrum. Given the genetic complexity of epilepsy, different genes cause specific epilepsy subtypes that are clinically indistinguishable and on the other hand, monogenic SNPs like in SCN1A cause varied phenotypes, from febrile seizure to epileptic encephalopathies. Therefore, this is the right time to proceed towards precision medicine in epilepsy. Apart from reducing the seizure frequency alone, a panoramic view of the disease mechanism with other factors like effect of common and rare variants, CNVs, polygenic risk evidences, pathways network, pharmacogenomics, other clinical phenotypes like neuro-images, EEG patterns, facilitate headway to precision medicine.

### 5.1. Prognosis

**Figure 4:**
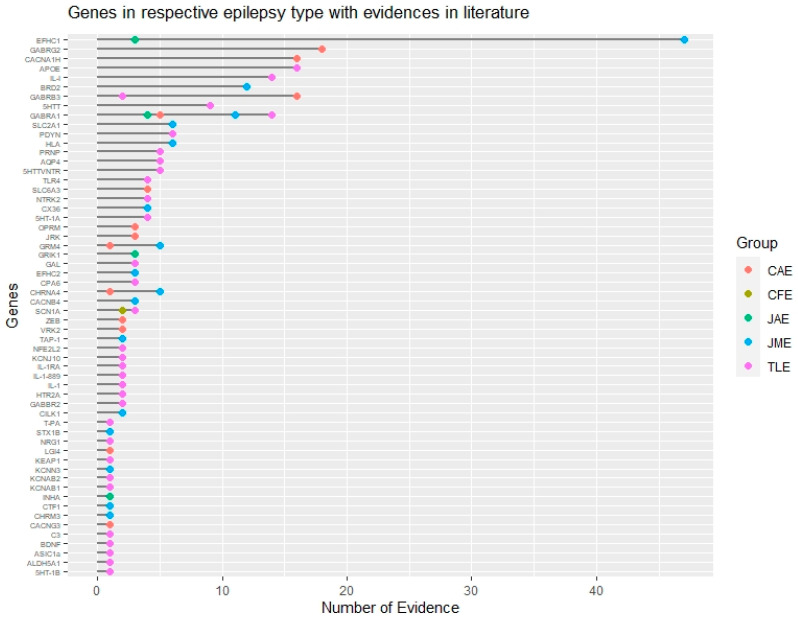
Genomic landscape of common epilepsy subtypes based on evidence. Genes associated with different subtypes of common epilepsies based on their literature evidence are represented by different color codes. This figure also shows common and non-overlapping genes associated with different epilepsy subtypes. CAE: Childhood absence epilepsy, CFE: Cryptogenic focal epilepsy, JAE: Juvenile absence epilepsy, JME: Juvenile myoclonic epilepsy, TLE: Temporal lobe epilepsy.

Variations in epilepsy genes generate variability in seizure type, epilepsy type, severities and other comorbidities. These variabilities may be due to single gene or a genetic variant (Figure 4). Genotyping along with deep phenotyping is essential in this genomic era. Genetic marker- specific prognosis is explored for rare epilepsy syndrome and is expanding for common epilepsies. It can render the diagnosis more certain in an early stage of the disease. For e.g. variations in KCNQ2 and KCNQ3 having 85% penetrance are identified as causal factor of benign familial neonatal seizures [258,259]. Genetic variants in such genes decrease the threshold membrane depolarization and increase neuronal burst [260]. Therefore, identification of variants in these genes with clinical phenotyping specific to benign familial neonatal seizures have a good prognosis, and can aid patient to get rid of seizures and medications at an early stage. But genetic variants in same genes cannot act as good biomarker for prognosis in all epileptic encephalopathy patient. In benign familial neonatal–infantile seizures patients, dominant point mutation is found in SCN2A gene, encode the alpha-subunit of the voltage-gated sodium channel NaV1.2 [261]. This point mutation results in increased neuronal excitability by gain of function [262,263]. Non-sense mutation found in the same gene result in more severe epilepsy and/or epilepsy encephalopathy, leading to a bad outcome. Thus, genetics can assist in outcome prediction in the benign familial epilepsies of childhood.

### 5.2. Diagnosis

Genetic insights into the disease have given a new direction to epilepsy diagnoses directly affecting clinical care. It not only controls seizure frequency but also improves neurodevelopmental comorbidities associated with the disease. It has the prospect of wider dispersal once new targeted treatments continue to emerge based on genetic evidences. Precision diagnostics is not new in the clinical management of epilepsies which follow Mendelian inheritance but progress to genetic analysis in common epilepsies is impeded by complex pattern of inheritance. However, limited findings of candidate gene studies and GWAS have suggested role of common variants in epilepsy, nonetheless all these are causal genes/variants necessarily be susceptible to disease risk. A genetic diagnosis is very important for disease management as it avoids the unnecessary repeated blood tests, invasive biopsies, MRIs, pre-surgical workup, and even unnecessary implementation of intracranial electrodes for monitoring electrical activity of seizure. Genetic diagnosis along with the family history is very helpful in estimating the epilepsy risk for other family members that are to be tested [264]. For example, studies have shown that polymorphisms in CACNA1H gene [25,265] GABAA receptor gene with variation γ2(R43Q, rs121909673) [39], β3(P11S; rs25409), β3(S15F; rs121913126), β3(G32R; rs71651682) [46], α1(S326fs328X) [95], GABRG2(IVS6 + 2T→G) [96], GABRB3 haplotype 2 are associated with CAE and these genes are commercially used for CAE genetic testing. Commercially available genetic testing is focused on gene panels that comprises individual gene, group of genes or chromosomal loci diagnosis a specific epileptic trait. Such tests exploit the advanced NGS platforms like whole genome sequencing, NGS or targeted sequencing or others. Targeted sequencing is used for identifying genetic variants in individual gene when specific epilepsy is suspected. Epilepsy gene panel involves the analysis of group of most common genes associated with discrete epilepsy sub-types. Advantage of using gene panel is that it covers all possible genetic cause of epilepsy. Chromosome microarray involves analysis of chromosome to check any imbalance that may cause epilepsy. Endo-phenotype markers along with genetics could be useful to dissect disease complexity. Imaging endo-phenotypes and genetics provide a link between brain features and underlying genetic architecture to facilitate identification of disease related genetic variants. For e.g., photo-peroxisomal EEG response is a common observation in JME and can be a useful endo-phenotype in epilepsy gene mapping. Candidate gene studies showed association of photo-peroxisomal EEG response with BRD2 gene in JME share some neurological pathways [266]. Motor system hyper-activation and impairment of memory is observed in JME patients and their siblings, implicating trait heritability and a JME endo-phenotype [267]. Alterations of temporal cortical surface area, absence of shared thickness abnormalities and varying patterns of hippocampal atrophy is detected in family studies of TLE patients [268]. Nowadays, machine learning through neuroimaging data (resting state functional MRI, diffusion tensor imaging is being used to find specific patterns in epilepsy, enabling seizure prediction, and to distinguish between active epilepsy patient and seizure free patient. Further, using clinical data, machine learning can also predict medical and surgical outcomes through using clinical data. One assessment of a support vector machine classifier revealed a peak diagnostic sensitivity of 82.5% and a specificity of 85% by evaluating the asymmetry of functional connectivity in homologous brain regions on resting-state functional MRI in 100 patients with epilepsy and 80 controls. These endo-phenotype studies will compliment for disease category phenotype studies [269,270]. There are number of genetic tests for rare epilepsies compared to common epilepsies. The genes available for genetic testing for common epilepsies are listed below in Table 3 (Supplementary Table S1) and for rare epilepsies are listed in Supplementary Table S2. Although making genetic diagnostics available to every patient is still critical and challenging. Several attempts made so far in the field of epilepsy genetic could be potential candidates that requires rigorous testing to prove safety and efficacy and demands appropriate functional validation before it is used for assessment in the clinic. Based on the literature evidence, diagnostic efficacy like sensitivity, specificity and positive predictive values are calculated for genetic variants are given in Table 4. These can be potential genetic markers for epilepsy testing.

### 5.3. Pharmacogenomics

The aim of pharmacogenomics is to predict how different individuals respond when prescribed with the same drug (and its dose). This aids in better clinical management of epilepsy by reducing adverse drug response and improving drug efficacy. Based on several such evidences, the USA food and drug administration (FDA) has approved drug labelling for patients with certain genetic variants. Most of them are addressed to reduce life-threatening adverse drug response. For e.g., the carriers of the rare POLG1 nucleotide substitution (p.Q1236H) may develop fatal hepatic failure when treated with sodium valproate [271]. A prospective genetic testing of such carrier patients may help clinicians identify individuals at high risk of this fatal drug toxicity. In other cases, one of the most widely studied HLA allele variant (HLA-B*15:02) predispose patients to a severe skin hypersensitivity (Stevens-Johnson syndrome/ toxic epidermal necrolysis) reaction when treated with carbamazepine [272]. It was investigated in Han Chinese population, and few other ethnic populations of Southeast Asian origin. On the contrary, HLA-A*3101 allele seemed to confer the risk of carbamazepine induced skin ADR in white people of northern Europe [273]. According to an alert from US FDA, Carbamazepine should be avoided if patients carry at least one copy of HLA-B*15:02 allele and if patients are having carbamazepine for few months and do not show any cutaneous reaction then such patients are at low risk of developing such reaction ever from carbamazepine FDA Alert [12/12/2007]. Hence, alternate drugs are recommended for patients with HLA-B*15:02 or HLA-A*3101 allele [274]. Screening for this allele prior to treatment administration may prove to be cost-effective as well as improve quality-of-life in people with epilepsy. Genetic testing for HLA-B*15:02 allele for predicting carbamazepine hypersensitivity, lamotrigine and phenytoin response is provided by HLA Laboratory Barnesin and by Millennium Health, USA.

A few studies also witnessed that genetic variants present in the drug metabolizing genes like CYP2C9, CYP2C19, drug transporters (ABCB1); or drug target gene SCN1A affect the binding of the drug to the receptor, and are responsible for altered drug efficacy. Around 90% metabolism of phenytoin is carried by CYP2C9 and rest 10% metabolism is carried by CYP2C19 [275]. Genetic polymorphism in CYP2C9 with variant allele *2 and *3 reduce phenytoin metabolism by 25–50% [275]. This results in increased susceptibility of those patients to phenytoin toxicity at usual administered doses. Contradictorily, one study in North Indian children showed that patients with alleles *2 and *3 had a significant increase in serum phenytoin, but without any adverse reaction [276]. Hence, CYP2C9 genotyping is done to rescue individuals for serious ADR from phenytoin. The Clinical Pharmacogenetics Implementation Consortium (CPIC) and FDA proposed that phenytoin should not be used for patients with CYP2C9 *2 and *3 variants and HLA-B*15:02 genotype [277]. Therefore, such studies help in improving the drug efficacy, alternate AED therapy administration or dosage change providing new therapeutic strategies in future. Such findings strongly establish the role of pharmacogenomics in better epilepsy treatment management and implementation in clinical settings for specific population. 

## 6. Conclusions and Future Direction

This is a new era for epilepsy genetics. Researchers and clinicians are joining hands, who are now swiftly moving towards evidence-based therapy for epilepsy management. Through the past decade, we have made remarkable progress towards gene discovery and innovation in technological and analytic determinants of this multi-faced disease. Once the gene attributing to the monogenic or polygenic cause of disease is clearly identified therapy can be targeted towards curing the defect contributed by the gene or compensate for the impaired molecular pathway caused by any variant of that gene. May be, that is why we have achieved much farther in monogenic epilepsies relative to its polygenic counterpart. From making progress towards identifying the cause of rare and severe epilepsy types to elucidating the role of molecular players deepens the understanding of patho-physiology of the disease. Anticipating the genetic variant type associated with epilepsy, its role in pathophysiology and quantitative assessment of the role of the genetic variant role in disease risk at individual and population level is a difficult challenge. It is causing a gap at translational level to implement genetics in targeted treatment. Hence, initiating curated registries of epilepsy patients, increasing number of multi-center, randomized, controlled trials are needed. Multi center collaboration like Epilepsy Genetic Initiative is providing a platform to bridge a gap between people with epilepsy, clinician and researchers for the advancement of precision medicine in epilepsy. This governs the way for epilepsy genetics into clinics. 

The future holds promise for progress in epilepsy genetic testing approaches that can be translated into improved disease diagnosis and treatment management for people with epilepsy. Large multi-national consortium and collaborative studies will generate huge data, which will be more valid and acceptable and help in making more accurate genotype–phenotype predictions. A panoramic approach is required for making advancement for precision medicine, which incorporate polygenic background and other non-genetic factors like microbiome, diet, optimal time for treatment, lifestyle like alcohol consumption and cigarette smoking which influence seizure threshold, sleep deprivation or stress should be considered which may enhance the success of the treatment [241].

A key focus is to develop a robust statistical genomic analysis approach that may consider the effect of variants in diverse population, demarcating the mutation patterns (allele frequency, its relative risk, and penetrance) contributing to the disease burden. This assures the application of genetics-based precision medicine in clinical settings. For this, it requires a multi-dimensional strategic design for effective treatment. A critical component in precision medicine would be integrating patient data obtained from multiple sources that includes genetic testing data, neuro-imaging data, biochemical profiling, comorbidities (e.g. cognitive, or psychiatric [278]), clinical and demographic details. Artificial intelligence and other technology can be used to exploit such multi-faced diseases for assessment of efficient outcomes. Developing algorithms exploiting artificial intelligence, which consider these subjective factors is inevitable. The findings and examples of genes and its variants associated with the common epilepsy phenotypes outlined in this review marks the translational potential of precision medicine into clinical care. This may guarantee the keystone for decision making in epilepsy therapy. In clinical management, genetics is potentially helping in epilepsy prognosis, diagnosis, opting a better treatment, provide provision for information regarding family planning, it is not confined to research realm only. 

Although significant findings have been observed in our study, several limitations exist. In this paper, though epilepsy has been broadly categorized into common and rare epilepsies based on their prevalence, the classification of common epilepsy subtypes are largely based on available literature evidences only. Additionally, most limitations of our study originate from the available genetic studies included. Owing to different factors contributing to study heterogeneity, like different sample size, study design, heterogeneous patient cohort, diagnostic criteria, reporting biases, may tamper the statistical significance of findings, accordingly. We have reported the significant genetic findings as per each paper included. No quality assessment or statistical test was performed to evaluate each study for the homogeneity or quantify the between-study variance. Further, all the genetic association p – values reported in Table 1, are unadjusted. There can be several co-factors which, along with the risk allele, may contribute to disease susceptibility. Such confounding factors have not been covered in our review. Sensitivity analyses are performed in our study to suggest possible markers that may be considered for commercial diagnostic management. However, risk alleles alone may not provide a robust evaluation of the disease risk, especially for complex disorders like epilepsy. One major limitation of genetic association studies is contributed by the genetic heterogeneity between studies because different markers in the same genes were employed for these associations; moreover, patients with different genetic background may not be strictly comparable. Genetic markers, if reported statistically non- significant, in replication cohort or in some other population, have not been reported in this article. The role of genetic variants in mitochondrial DNA associated with epilepsy was also not been covered here. Our review is limited to studies identified only from MEDLINE (or PubMed).

## Figures and Tables

**Table 2:** Different copy number variants (CNVs) associated with epilepsies.

Sl. No.	Locus	Size	CNVs	Gene		Phenotype	Consequences	Country	Year	References
1	2p16.3	~287 and ~79 kb	Exon-disrupting deletion		*Nrxn1*	Severe early onset epilepsy	Alteration in the calcium-dependent release of neurotransmitters	UK	2011 (case report)	[251]
2	2q24.2-q24.3	11Mb	Duplication/deletion		*SCN1A*, *SCN2A*, *SLC4A10*	Idiopathic epilepsy	Imbalance in sodium channel	Brazil	2010 (case report)	[252]
3	6p12.1	99.9kb	Micro-duplication		*BMP5*	Epilepsy	Increased cell death in ventral forebrain	Saudi Arabia	2015	[253]
4	7q11.22	78.7kb	Deletion		*AUTS2*	JME	Role in neurodevelopment	Switzerland, Germany, USA	2010	[254]
5	7q32.3	63.9kb	Microdeletion		*PODXL*	Epilepsy	Malignant progression of astrocytic tumors	Saudi Arabia	2015	[253]
6	7q35	785.8kb	Deletion, hemizygous deletions		*CNTNAP2*	GTCS	Affects cell–cell interaction in nervous system	Switzerland, Germany, USA	2010	[254]
7	15q11.2		Microdeletion		*NIPA2, CYFIP1*	GGE	Role in neuronal growth and differentiation	Austria, Belgium, Denmark, Germany, the Netherlands	2010	[246]
8	15q13.3	1.4Mb	Microdeletion		*CHRNA7*	GGE (specifically JME)	Modulates thalamo-cortical pathways	Switzerland, Germany, USA	2009, 2010	[244,254]
9	16p13.11	800Kb	Deletion		*NDE1*	MTLE	Brain structural alterations	UK	2012	[255]
10	16p13.2	47Kb	Microdeletion		*GRIN2A*	RE (childhood epilepsy)	Disrupt *GRIN2A*	France	2014	[256]
11	22q11.2	3Mb	Microdeletion		*DGCR6, DGCR6L*	GGE (specifically JME)	Haplo-insufficiency of DGCR6 in 22q11 could disturb interaction with GABA_B_R	The Netherlands	2016	[257]

**Table 3:** List of available genetic diagnosis markers for common epilepsies.

Sl. No.	Gene	Genomic Loci	Putative Markers	Gene Function	Phenotype Prediction	Company	Major Ethnic Group
1	*SLC2A1*	1p34.2	-	Solute carrier transporter	Genetic generalized epilepsy	Centogene AG the Rare Disease Company and Blueprint Genetics	European
2	*CACNB4*	2q23.3	-	Voltage-gated calcium channel			
3	*GABRG2*	5q34	-	GABA receptor	Generalized epilepsy with febrile seizures plus	Invitae, GeneDx, Fulgent Genetics, LifeLabs Genetics, Laboratoria de Genetica Clinica SL, Institute of Human Genetics and Cologne University	American and European
4	*STX1B*	16p11.2	-	Synaptic vesicle			
5	*SCN1A*	2q24.3	rs8191987, rs16851381, rs2298771	Voltage-dependent sodium channel			
6	*SCN1B*	19q13.11		Voltage-gated sodium channel			
7	*SCN9A*	2q24.3		Voltage-gated sodium channel			
8	*GABRA1*	5q34	rs1581220270	GABA receptor	Childhood absence epilepsy	Invitae, LifeLabs Genetics and Clinical Molecular Genetics Laboratory	American
9	*GABRB3*	15q12	rs25409	Ligand-gated ionic channel			
10	*GABRG2*	5q34	rs1561645243	GABA receptor			
11	*JRK*	8q24.3	T456M	DNA-binding protein			
12	*CACNA1H*	16p13.3	rs9934839, rs2745150, rs8044363, rs8043905, rs9934839, rs3751664, c.937A>G, rs119454947, rs119454949	Voltage-dependent calcium channel			
13	*CACNB4*	2q23.3	R482X	Voltage-dependent calcium channel	Juvenile myoclonic epilepsy	Athena Diagnostics Inc, GeneDx, Invitae, MedGen and Illumina Clinical Services Laboratory	American & European
14	*EFHC1*	6p12.2	rs137852778, rs137852776, rs137852777, rs149055334, rs79761183	Calcium-binding protein			
15	*GABRA1*	5q34	rs121434579	GABA receptor			
16	*CILK1*	6p12.1	rs376111440, rs55932059, rs1554169267, rs765078446	Protein kinases			
17	*GAL*	11q13.2	C116A	Galanin and GMAP prepropeptide	Familial temporal lobe epilepsy	Invitae, GeneDx, Fulgent Genetics, LifeLabs Genetics, Laboratoria de Genetica Clinica SL, Institute of Human Genetics, Cologne University, Athena Diagnostics Inc, CGC Genetics, MNG Laboratories (Medical Neurogenetics, LLC), PreventionGenetics, Amplexa Genetics	American & European
18	*CPA6*	8q13.2	rs114402678, rs61738009	Metallocarboxypeptidases			
19	*RELN*	7q22.1	-	Cell–cell interaction			
20	*LGI1*	10q23.33	-	-			
21	*MICAL1*	6q21	-	Depolymerization of actin filaments			

In this table, we have mentioned the genes that are used for genetic testing by different testing companies. These genes are obtained from the Genetic Testing Registry (GTR).

**Table 4:** Genetic marker for genetic testing in epilepsy with diagnostic efficacy.

Sl No	Gene	Variant	Location	Risk Allele	GMAF	Phenotype	Sample Size (P/C)	Country	Study (PMID)	Sensitivity	Specificity	PPV	OR
1.	*Cx36*	rs3743123	Coding	T	0.401	JME	169/123	UK, Denmark, France, Greece, Portugal, and Sweden	15235036	37.8	70.7	37.4	1.4
							247/621	Germany	16876983				
2.	*SCN1A*	rs3812718	Intronic	A	0.581	Epilepsy	76/701	Australia	19949041	56.6	47.6	39.4	1.2
							90/701		19289736				
							97/837	China	20477842				
							362/86	India	20602612				
							62/199	Switzerland	21762453				
							138/282	India	22578703				
							234/189	Taiwan	22188362				
							485/298	India	23466530				
							133/209	Austria	24014518				
							212/344	German					
							282/470	Malaysian Chinese	25668517				
							151/244	India					
							243/358	Malaysia					
							200/200	Greece	28144265				
3.	*GABRG2*	rs211037	Coding	T	0.222	Epilepsy	135/154	Germany	12117362	32.6	45.1	38.5	0.6
							53/96	Italy	12694927				
							104/83	Taiwan	12672902				
							94/106	Japan	12759178				
							569/330	British	16806831				
							684/284	Ireland					
							74/118	America	16256272				
							77/83	Taiwan	17162195				
							100/120	Egypt	21983990				
							441/267	India	24061200				
							60/153	Roman	29379546				
							100/100	Brazil	23287319				
							1719/4672	Malaysia, Hong Kong, Korea, India	26452361				
4	*BRD2*	rs3918149	5′ UTR	A	0.246	JME	531/1390	Britain, Iran, Germany, Australia, India	17437413	17	13	27.4	1.14
							116/470	Germany	30719712				
5	*CACNA1H*	rs9934839	Coding	G	0.340	CAE	100/191	China	16905256	9.17	5.49	65.6	1.73
							218/191	China	17156077				

GMAF: Global minor allele frequency.

## Supplementary Materials

Supplementary materials can be found at https://www.mdpi.com/1422-0067/21/20/7784/s1, Table S1: List of gene panels used for epilepsy diagnosis in different companies. Table S2: List of available genes used for genetic diagnosis of rare epilepsies.

## References

[1] WHO (2020). Epilepsy Factsheet. WHO Webpage. https://www.who.int/news-room/fact-sheets/detail/epilepsy.

[2] Beghi E. (2019). The Epidemiology of Epilepsy. Neuroepidemiology.

[3] Gibson G. (2011). Rare and common variants: Twenty arguments. Nat. Rev. Genet.

[4] Buono R.J. (2013). Genome wide association studies (GWAS) and common forms of human epilepsy. Epilepsy Behav..

[5] Weber Y.G., Lerche H. (2008). Genetic mechanisms in idiopathic epilepsies. Dev. Med. Child Neurol..

[6] Prasad D.K.V., Satyanarayana U., Munshi A. (2013). Genetics of idiopathic generalized epilepsy: An overview. Neurol. India.

[7] Lennox W.G. (1951). The heredity of epilepsy as told by relatives and twins. J. Am. Med. Assoc..

[8] Myers C.T., Mefford H.C. (2015). Advancing epilepsy genetics in the genomic era. Genome Med..

[9] Alhusaini S., Whelan C.D., Sisodiya S.M., Thompson P.M. (2016). Quantitative magnetic resonance imaging traits as endophenotypes for genetic mapping in epilepsy. Neuroimage Clin..

[10] Gottesman I.I., Gould T.D. (2003). The Endophenotype Concept in Psychiatry: Etymology and Strategic Intentions. Am. J. Psychiatry.

[11] Kasperavičiūtė D., Catarino C.B., Heinzen E.L., Depondt C., Cavalleri G.L., Caboclo L.O., Tate S.K., Jamnadas-Khoda J., Chinthapalli K., Clayton L.M. (2010). Common genetic variation and susceptibility to partial epilepsies: A genome-wide association study. Brain.

[12] Guo Y., Baum L.W., Sham P.C., Wong V., Ng P.W., Lui C.H.T., Sin N.C., Tsoi T.H., Tang C.S., Kwan J.S. (2011). Two-stage genome-wide association study identifies variants in CAMSAP1L1 as susceptibility loci for epilepsy in Chinese. Hum. Mol. Genet..

[13] (2018). The International League against Epilepsy Consortium on Complex Epilepsies Genome-wide mega-analysis identifies 16 loci and highlights diverse biological mechanisms in the common epilepsies. Nat. Commun..

[14] Scheffer I.E., Berkovic S., Meletti S., Connolly M.B., French J., Guilhoto L., Hirsch E., Jain S., Mathern G.W., Moshé S.L. (2017). ILAE classification of the epilepsies: Position paper of the ILAE Commission for Classification and Terminology. Epilepsia.

[15] Commission on Classification and Terminology of the International League against Epilepsy (1989). Proposal for revised classification of epilepsies and epileptic syndromes. Commission on Classification and Terminology of the International League against Epilepsy. Epilepsia.

[16] Wallace R.H., Wang D.W., Singh R., Scheffer I.E., George A.L., Phillips H.A., Saar K., Reis A., Johnson E.W., Sutherland G.R. (1998). Febrile seizures and generalized epilepsy associated with a mutation in the Na+-channel ß1 subunit gene SCN1B. Nat. Genet..

[17] McNamara J.O. (1999). Emerging insights into the genesis of epilepsy. Nature.

[18] Steinlein O.K. (2004). Genetic mechanisms that underlie epilepsy. Nat. Rev. Neurosci..

[19] Staley K. (2015). Molecular mechanisms of epilepsy. Nat. Neurosci..

[20] Mullen S.A., Berkovic S.F., Commission T.I.G. (2018). Genetic generalized epilepsies. Epilepsia.

[21] Sander T., Schulz H., Saar K., Gennaro E., Riggio M.C., Bianchi A., Zara F., Luna D., Bulteau C., Kaminska A. (2000). Genome search for susceptibility loci of common idiopathic generalised epilepsies. Hum. Mol. Genet..

[22] Crunelli V., LeResche N. (2002). Childhood absence epilepsy: Genes, channels, neurons and networks. Nat. Rev. Neurosci..

[23] Foundation E. Childhood Absence Epilepsy. https://www.epilepsy.com/learn/types-epilepsy-syndromes/childhood-absence-epilepsy.

[24] Özlem Y. (2012). Genes and molecular mechanisms involved in the epileptogenesis of idiopathic absence epilepsies. Seizure.

[25] Liang J., Zhang Y., Chen Y., Wang J., Pan H., Wu H., Xu K., Liu X., Jiang Y., Shen Y. (2006). Common Polymorphisms in theCACNA1HGene Associated with Childhood Absence Epilepsy in Chinese Han Population. Ann. Hum. Genet..

[26] Everett K., Chioza B.A., Aicardi J., Aschauer H.N., Brouwer O., Callenbach P.M., Covanis A., Dooley J., Dulac O., Durner M. (2007). Linkage and mutational analysis of CLCN2 in childhood absence epilepsy. Epilepsy Res..

[27] Oyrer J., Maljevic S., Scheffer I.E., Berkovic S.F., Petrou S., Reid C.A. (2017). Ion Channels in Genetic Epilepsy: From Genes and Mechanisms to Disease-Targeted Therapies. Pharm. Rev..

[28] Alexander S.P., Fabbro D., Kelly E., Marrion N., Peters J.A., Benson H.E., Faccenda E., Pawson A.J., Sharman J.L., Southan C. (2015). The Concise Guide to Pharmacology 2015/16: Enzymes. Br. J. Pharm..

[29] Feng T., Kalyaanamoorthy S., Barakat K. (2018). L-Type Calcium Channels: Structure and Functions. Ion Channels Health Sick..

[30] Chen Y., Lu J., Pan H., Zhang Y., Wu H., Xu K., Liu X., Jiang Y., Bao X., Yao Z. (2003). Association between genetic variation ofCACNA1H and childhood absence epilepsy. Ann. Neurol..

[31] Liang J., Zhang Y., Wang J., Pan H., Wu H., Xu K., Liu X., Jiang Y., Shen Y., Wu X.-R. (2006). New variants in the CACNA1H gene identified in childhood absence epilepsy. Neurosci. Lett..

[32] Khosravani H., Altier C., Simms B., Hamming K.S., Snutch T.P., Mezeyova J., McRory J.E., Zamponi G.W. (2004). Gating Effects of Mutations in the Cav3.2 T-type Calcium Channel Associated with Childhood Absence Epilepsy. J. Biol. Chem..

[33] Meldrum B.S. (1994). The role of glutamate in epilepsy and other CNS disorders. Neurology.

[34] Rogawski M.A., Donevan S.D. (1999). AMPA receptors in epilepsy and as targets for antiepileptic drugs. Adv. Neurol..

[35] Rogawski M.A. (2013). AMPA receptors as a molecular target in epilepsy therapy. Acta Neurol. Scand. Suppl..

[36] Gallagher M.J., Song L., Arain F., Macdonald R.L. (2004). The Juvenile Myoclonic Epilepsy GABAA Receptor 1 Subunit Mutation A322D Produces Asymmetrical, Subunit Position-Dependent Reduction of Heterozygous Receptor Currents and 1 Subunit Protein Expression. J. Neurosci..

[37] Ewatanabe M., Efukuda A. (2015). Development and regulation of chloride homeostasis in the central nervous system. Front. Cell. Neurosci..

[38] Bowser D.N., Wagner D.A., Czajkowski C., Cromer B.A., Parker M.W., Wallace R.H., Harkin L.A., Mulley J.C., Marini C., Berkovic S.F. (2002). Altered kinetics and benzodiazepine sensitivity of a GABAA receptor subunit mutation [ 2(R43Q)] found in human epilepsy. Proc. Natl. Acad. Sci. USA.

[39] Wallace R.H., Marini C., Petrou S., Harkin L.A., Bowser D.N., Panchal R.G., Williams D.A., Sutherland G.R., Mulley J.C., Scheffer I.E. (2001). Mutant GABA A receptor γ2-subunit in childhood absence epilepsy and febrile seizures. Nat. Genet..

[40] Sancar F., Czajkowski C. (2004). A GABAA receptor mutation linked to human epilepsy (γ2R43Q) impairs cell surface expression of αβγ receptors. J. Biol. Chem..

[41] Kang J., Macdonald R.L. (2004). The GABAA Receptor 2 Subunit R43Q Mutation Linked to Childhood Absence Epilepsy and Febrile Seizures Causes Retention of 1 2 2S Receptors in the Endoplasmic Reticulum. J. Neurosci..

[42] Frugier G., Coussen F., Emerit M.B., Garret M., Giraud M.-F., Odessa M.-F., Boué-Grabot É. (2006). A γ2(R43Q) Mutation, Linked to Epilepsy in Humans, Alters GABAA Receptor Assembly and Modifies Subunit Composition on the Cell Surface*. J. Biol. Chem..

[43] Chaumont S., André C., Perrais D., Boué-Grabot É., Taly A., Garret M. (2013). Agonist-dependent Endocytosis of γ-Aminobutyric Acid Type A (GABAA) Receptors Revealed by a γ2(R43Q) Epilepsy Mutation. J. Biol. Chem..

[44] Feucht M., Fuchs K., Pichlbauer E., Hornik K., Scharfetter J., Goessler R., Füreder T., Cvetkovic N., Sieghart W., Kasper S. (1999). Possible association between childhood absence epilepsy and the gene encoding GABRB3. Biol. Psychiatry.

[45] Urak L., Feucht M., Fathi N., Hornik K., Fuchs K. (2006). A GABRB3 promoter haplotype associated with childhood absence epilepsy impairs transcriptional activity. Hum. Mol. Genet..

[46] Tanaka M., Olsen R.W., Medina M.T., Schwartz E., Alonso M.E., Durón R.M., Castro-Ortega R., Martinez-Juarez I.E., Pascual-Castroviejo I., Machado-Salas J. (2008). Hyperglycosylation and Reduced GABA Currents of Mutated GABRB3 Polypeptide in Remitting Childhood Absence Epilepsy. Am. J. Hum. Genet..

[47] Delahanty R.J., Kang J.Q., Brune C.W., Kistner E.O., Courchesne E., Cox N.J., Cook E.H., Macdonald R.L., Sutcliffe J.S. (2009). Maternal transmission of a rare GABRB3 signal peptide variant is associated with autism. Mol. Psychiatry.

[48] Thomsen C., Dalby N.O. (1998). Roles of metabotropic glutamate receptor subtypes in modulation of pentylenetetrazole-induced seizure activity in mice. Neuropharmacology.

[49] Ngomba R., Ferraguti F., Badura A., Citraro R., Santolini I., Battaglia G., Bruno V., De Sarro G., Simonyi A., Van Luijtelaar G. (2008). Positive allosteric modulation of metabotropic glutamate 4 (mGlu4) receptors enhances spontaneous and evoked absence seizures. Neuropharmacology.

[50] Snead O.C., Banerjee P.K., Burnham M., Hampson D. (2000). Modulation of Absence Seizures by the GABAA Receptor: A Critical Role for Metabotropic Glutamate Receptor 4 (mGluR4). J. Neurosci..

[51] Muhle H., Von Spiczak S., Gaus V., Kara S., Helbig I., Hampe J., Franke A., Weber Y., Lerche H., Kleefuss-Lie A.A. (2010). Role of GRM4 in idiopathic generalized epilepsies analysed by genetic association and sequence analysis. Epilepsy Res..

[52] Steinlein O., Sander T., Stoodt J., Kretz R., Janz D., Propping P. (1997). Possible association of a silent polymorphism in the neuronal nicotinic acetylcholine receptor subunit α4 with common idiopathic generalized epilepsies. Am. J. Med Genet..

[53] Vaccarino A.L., Olson G.A., Olson R.D., Kastin A.J. (1999). Endogenous opiates: 1998. Peptides.

[54] Lasoń W., Przewłocka B., Coenen A., Przewłocki R., Van Luijtelaar G. (1994). Effects of μ and δ opioid receptor agonists and antagonists on absence epilepsy in WAG/Rij rats. Neuropharmacology.

[55] Przewłocka B., Lasoń W., Turchan J., De Bruin N.M.W.J., Van Luijtelaar G., Przewłocki R., Coenen A. (1998). Anatomical and functional aspects of μ opioid receptors in epileptic WAG/Rij rats. Epilepsy Res..

[56] Dua A.K., Pinsky C., Labella F.S. (1985). MU- and Delta-opioid receptor-mediated epileptoid responses in morphine-dependent and non-dependent rats. Electroencephalogr. Clin. Neurophysiol..

[57] Sander T., Berlin W., Gscheidel N., Wendel B., Janz D., Hoehe M.R. (2000). Genetic variation of the human μ-opioid receptor and susceptibility to idiopathic absence epilepsy. Epilepsy Res..

[58] Barratt C., Lai T., Fisniku L., Moran N., Nashef L., Valentin A., Asherson P., Makoff A. (2006). No Association of Single Nucleotide Polymorphisms in the Opioid Receptor Subunit Gene with Idiopathic Generalized Epilepsy. Epilepsia.

[59] Sander T., Berlin W., Ostapowicz A., Samochowiec J., Gscheidel N., Hoehe M. (2000). Variation of the genes encoding the human glutamate EAAT2, serotonin and dopamine transporters and Susceptibility to idiopathic generalized epilepsy. Epilepsy Res..

[60] Sander T., Harms H., Podschus J., Finckh U., Nickel B., Rolfs A., Rommelspacher H., Schmidt L.G. (1997). Allelic association of a dopamine transporter gene polymorphism in alcohol dependence with withdrawal seizures or delirium. Biol. Psychiatry.

[61] Szot P., Reigel C.E., White S.S., Veith R.C. (1996). Alterations in mRNA expression of systems that regulate neurotransmitter synaptic content in seizure-naive genetically epilepsy-prone rat (GEPR): Transporter proteins and rate-limiting synthesizing enzymes for norepinephrine, dopamine and serotonin. Mol. Brain Res..

[62] Gu W., Sander T., Becker T., Steinlein O.K. (2004). Genotypic association of exonic LGI4 polymorphisms and childhood absence epilepsy. Neurogenetics.

[63] Genton P., Thomas P., Trenité D.G.K.-N., Medina M.T., Salas-Puig J. (2013). Clinical aspects of juvenile myoclonic epilepsy. Epilepsy Behav..

[64] Gilsoul M., Grisar T., Delgado-Escueta A.V., De Nijs L., Lakaye B. (2019). Subtle Brain Developmental Abnormalities in the Pathogenesis of Juvenile Myoclonic Epilepsy. Front. Cell. Neurosci..

[65] Striano P., Nobile C. (2018). The genetic basis of juvenile myoclonic epilepsy. Lancet Neurol..

[66] Delgado-Escueta A.V. (2007). Advances in genetics of juvenile myoclonic epilepsies. Epilepsy Curr..

[67] Dos Santos B.P., Marinho C.R.M., Marques T.E.B.S., Angelo L.K.G., Malta M.V.D.S., Duzzioni M., De Castro O.W., Leite J.P., Barbosa F.T., Gitaí D.L.G. (2017). Genetic susceptibility in Juvenile Myoclonic Epilepsy: Systematic review of genetic association studies. PLoS ONE.

[68] Medina M.T., Duron R., Alonso M.E. (2005). Novel mutations in myoclonin1/EFHC1 in families from Honduras, Mexico and Japan. Epilepsia.

[69] Ma S., Blair M.A., Abou-Khalil B., Lagrange A.H., Gurnett C.A., Hedera P. (2006). Mutations in the GABRA1 and EFHC1 genes are rare in familial juvenile myoclonic epilepsy. Epilepsy Res..

[70] De Nijs L., Wolkoff N., Coumans B., Delgado-Escueta A.V., Grisar T., Lakaye B. (2012). Mutations of EFHC1, linked to juvenile myoclonic epilepsy, disrupt radial and tangential migrations during brain development. Hum. Mol. Genet..

[71] Noctor S.C., Flint A.C., Weissman T.A., Dammerman R.S., Kriegstein A.R. (2001). Neurons derived from radial glial cells establish radial units in neocortex. Nat. Cell Biol..

[72] Woermann F.G., Free S.L., Koepp M.J., Sisodiya S.M., Duncan J.S. (1999). Abnormal cerebral structure in juvenile myoclonic epilepsy demonstrated with voxel-based analysis of MRI. Brain.

[73] De Nijs L., Leon C., Nguyen L., LoTurco J.J., Delgado-Escueta A.V., Grisar T., Lakaye B. (2009). EFHC1 interacts with microtubules to regulate cell division and cortical development. Nat. Neurosci..

[74] Neubauer B.A., Waldegger S., Heinzinger J., Hahn A., Kurlemann G., Fiedler B., Eberhard F., Muhle H., Stephani U., Garkisch S. (2008). KCNQ2 and KCNQ3 mutations contribute to different idiopathic epilepsy syndromes. Neurology.

[75] Sander T., Toliat M.R., Heils A., Leschik G., Becker C., Rüschendorf F., Rohde K., Mundlos S., Nürnberg P. (2002). Association of the 867Asp variant of the human anion exchanger 3 gene with common subtypes of idiopathic generalized epilepsy. Epilepsy Res..

[76] Balan S., Vellichirammal N.N., Banerjee M., Radhakrishnan K. (2012). Failure to find association between febrile seizures and SCN1A rs3812718 polymorphism in south Indian patients with mesial temporal lobe epilepsy and hippocampal sclerosis. Epilepsy Res..

[77] Lee C.-C., Chou I.-C., Tsai C.-H., Wan L., Shu Y.-A., Tsai Y., Lin C.-C., Tsai F.-J. (2007). Association of idiopathic generalized epilepsy with polymorphisms in the neuronal nicotinic acetylcholine receptor subunits. J. Clin. Lab. Anal..

[78] Lucarelli P., Rizzo R., Gagliano A., Palmarino M., Volzone A., Arpino C., Curatolo P. (2007). Association between D18S474 locus on chromosome 18q12 and idiopathic generalized epilepsy. Brain Dev..

[79] Prasad D., Shaheen U., Satyanarayana U., Prabha T.S., Jyothy A., Munshi A. (2014). Association of GABRA6 1519 T > C (rs3219151) and Synapsin II (rs37733634) gene polymorphisms with the development of idiopathic generalized epilepsy. Epilepsy Res..

[80] El Ella S.S.A., Tawfik M.A., El-Fotoh W.M.M.A., Soliman O.A.M. (2018). The genetic variant “C588T” of GABARG2 is linked to childhood idiopathic generalized epilepsy and resistance to antiepileptic drugs. Seizure.

[81] Sander T., Hildmann T., Kretz R., Fürst R., Sailer U., Bauer G., Schmitz B., Beck-Mannagetta G., Wienker T.F., Janz D. (1997). Allelic association of juvenile absence epilepsy with a GluR5 kainate receptor gene (GRIK1) polymorphism. Am. J. Med Genet..

[82] Al-Eitan L., Al-Dalalah I.M., Aljamal H. (2019). Effects of GRM4, SCN2A and SCN3B polymorphisms on antiepileptic drugs responsiveness and epilepsy susceptibility. Saudi Pharm. J..

[83] Gloria-Bottini F., Lucarelli P., Saccucci P., Cozzoli E., Cerminara C., Curatolo P., Bottini E. (2008). Genetic Polymorphism and Idiopathic Generalized Epilepsy. Evidence of Interaction between Haptoglobin and ACP1 Systems. Neuropediatrics.

[84] Chioza B.A., Osei-Lah A., Wilkie H., Nashef L., McCormick D., Asherson P., Makoff A. (2002). Suggestive evidence for association of two potassium channel genes with different idiopathic generalised epilepsy syndromes. Epilepsy Res..

[85] Lenzen K., Heils A., Lorenz S., Hempelmann A., Hofels S., Lohoff F., Schmitz B., Sander T. (2005). Supportive evidence for an allelic association of the human KCNJ10 potassium channel gene with idiopathic generalized epilepsy. Epilepsy Res..

[86] Lorenz S., Heils A., Kasper J.M., Sander T. (2006). Allelic association of a truncation mutation of theKCNMB3 gene with idiopathic generalized epilepsy. Am. J. Med. Genet. Part B Neuropsychiatr. Genet..

[87] Lakhan R., Kalita J., Misra U., Kumari R., Mittal B. (2010). Association of intronic polymorphism rs3773364 A>G in synapsin-2 gene with idiopathic epilepsy. Synapse.

[88] Dean J., Robertson Z., Reid V., Wang Q., Hailey H., Moore S., Rasalam A.D., Turnpenny P., Lloyd D., Shaw D. (2008). A high frequency of the MTHFR 677C>T polymorphism in Scottish women with epilepsy: Possible role in pathogenesis. Seizure.

[89] Epicure C., Steffens M., Leu C., Ruppert A.-K., Zara F., Striano S., Robbiano A., Capovilla G., Tinuper P., Gambardella A. (2012). Genome-wide association analysis of genetic generalized epilepsies implicates susceptibility loci at 1q43, 2p16.1, 2q22.3 and 17q21.32. Hum. Mol. Genet..

[90] Makoff A., Lai T., Barratt C., Valentin A., Moran N., Asherson P., Nashef L. (2010). High-density SNP screen of sodium channel genes by haplotype tagging and DNA pooling for association with idiopathic generalized epilepsy. Epilepsia.

[91] Kahle K.T., Merner N.D., Friedel P., Silayeva L., Liang B., Khanna A., Shang Y., Lachance-Touchette P., Bourassa C., Levert A. (2014). Genetically encoded impairment of neuronal KCC 2 cotransporter function in human idiopathic generalized epilepsy. EMBO Rep..

[92] Layouni S., Chouchane L., Malafosse A., Dogui M. (2010). Dimorphism ofTAP-1gene in Caucasian with juvenile myoclonic epilepsy and in Tunisian with idiopathic generalized epilepsies. Int. J. Immunogenet..

[93] Striano P., Weber Y.G., Toliat M.R., Schubert J., Leu C., Chaimana R., Baulac S., Guerrero R., LeGuern E., Lehesjoki A.-E. (2012). GLUT1 mutations are a rare cause of familial idiopathic generalized epilepsy. Neurology.

[94] (2014). International League against Epilepsy Consortium on Complex Epilepsies Genetic determinants of common epilepsies: A meta-analysis of genome-wide association studies. Lancet Neurol..

[95] Maljevic S., Krampfl K., Cobilanschi J., Tilgen N., Beyer S., Weber Y.G., Schlesinger F., Ursu D., Melzer W., Cossette P. (2006). A mutation in the GABAAreceptor α1-subunit is associated with absence epilepsy. Ann. Neurol..

[96] Kananura C., Haug K., Sander T., Runge U., Gu W., Hallmann K., Rebstock J., Heils A., Steinlein O.K. (2002). A Splice-Site Mutation in GABRG2 Associated With Childhood Absence Epilepsy and Febrile Convulsions. Arch. Neurol..

[97] Moore T., Hecquet S., McLellann A., Ville D., Grid D., Picard F., Moulard B., Asherson P., Makoff A., McCormick D. (2001). Polymorphism analysis of JRK/JH8, the human homologue of mouse jerky, and description of a rare mutation in a case of CAE evolving to JME. Epilepsy Res..

[98] Cavalleri G.L., Walley N.M., Soranzo N., Mulley J., Doherty C.P., Kapoor A., Depondt C., Lynch J.M., Scheffer I.E., Heils A. (2007). A Multicenter Study of BRD2 as a Risk Factor for Juvenile Myoclonic Epilepsy. Epilepsia.

[99] Pal D.K., Evgrafov O.V., Tabares P., Zhang F., Durner M., Greenberg D.A. (2003). BRD2 (RING3) Is a Probable Major Susceptibility Gene for Common Juvenile Myoclonic Epilepsy. Am. J. Hum. Genet..

[100] Rozycka A., Steinborn B., Trzeciak W.H. (2009). The 1674+11C>T polymorphism of CHRNA4 is associated with juvenile myoclonic epilepsy. Seizure.

[101] Escayg A., De Waard M., Lee D.D., Bichet D., Wolf P., Mayer T., Johnston J., Baloh R., Sander T., Meisler M.H. (2000). Coding and Noncoding Variation of the Human Calcium-Channel β4-Subunit Gene CACNB4 in Patients with Idiopathic Generalized Epilepsy and Episodic Ataxia. Am. J. Hum. Genet..

[102] Bailey J.N., De Nijs L., Bai D., Suzuki T., Miyamoto H., Tanaka M., Patterson C., Lin Y.-C., Medina M.T., Alonso M.E. (2018). Variant Intestinal-Cell Kinase in Juvenile Myoclonic Epilepsy. N. Engl. J. Med..

[103] Hempelmann A., Heils A., Sander T. (2006). Confirmatory evidence for an association of the connexin-36 gene with juvenile myoclonic epilepsy. Epilepsy Res..

[104] Mas C., Taske N., Deutsch S., Guipponi M., Thomas P., Covanis A., Friis M., Kjeldsen M.J., Pizzolato G.P., Villemure J.-G. (2004). Association of the connexin36 gene with juvenile myoclonic epilepsy. J. Med. Genet..

[105] Suzuki T., Miyamoto H., Nakahari T., Inoue I., Suemoto T., Jiang B., Hirota Y., Itohara S., Saido T.C., Tsumoto T. (2009). Efhc1 deficiency causes spontaneous myoclonus and increased seizure susceptibility. Hum. Mol. Genet..

[106] Gu W., Sander T., Heils A., Lenzen K.P., Steinlein O.K. (2005). A new EF-hand containing gene EFHC2 on Xp11.4: Tentative evidence for association with juvenile myoclonic epilepsy. Epilepsy Res..

[107] Walz R., Castro R.M., Velasco T.R., Alexandre V., Lopes M.H., Leite J.P., Santos A.C., Assirati J.A., Wichert-Ana L., Terra-Bustamante V.C. (2003). Surgical outcome in mesial temporal sclerosis correlates with prion protein gene variant. Neurology.

[108] Parihar R., Mishra R., Singh S.K., Jayalakshmi S., Mehndiratta M.M., Ganesh S. (2014). Association of the GRM4 gene variants with juvenile myoclonic epilepsy in an Indian population. J. Genet..

[109] Greenberg D.A., Durner M., Shinnar S., Resor S., Rosenbaum D., Klotz I., Dicker E., Keddache M., Zhou G., Yang X. (1996). Association of HLA class II alleles in patients with juvenile myoclonic epilepsy compared with patients with other forms of adolescent-onset generalized epilepsy. Neurology.

[110] Yamamoto S., Yamamoto J., Kotani K., Shimizu A. (1995). A study of the association between Japanese juvenile myoclonic epilepsy patients and HLA class II antigens. Psychiatry Clin. Neurosci..

[111] Obeid T., Rab M.O.G., Daif A.K., Panayiotopoulos C.P., Halim K., Bahakim H., Bamgboye E. (1994). Is HLA-DRW 13 (W6) Associated with Juvenile Myoclonic Epilepsy in Arab Patients?. Epilepsia.

[112] Layouni S., Buresi C., Thomas P., Malafosse A., Dogui M. (2009). BRD2 and TAP-1 genes and juvenile myoclonic epilepsy. Neurol. Sci..

[113] Yalcin O., Baykan B., Agan K., Yapici Z., Yalçın D., Dizdarer G., Turkdogan D., Özkara Ç., Ünalp A., Uluduz D. (2011). An association analysis at 2q36 reveals a new candidate susceptibility gene for juvenile absence epilepsy and/or absence seizures associated with generalized tonic-clonic seizures. Epilepsia.

[114] Briellmann R.S., Torn-Broers Y., Busuttil B.E., Major B.J., Kalnins R.M., Olsen M., Jackson G.D., Frauman A.G., Berkovic S.F. (2000). APOE 4 genotype is associated with an earlier onset of chronic temporal lobe epilepsy. Neurol..

[115] Yeni S.N., Ozkara C., Buyru N., Baykara O., Hanoğlu L., Karaagac N., Ozyurt E., Uzan M. (2005). Association between APOE polymorphisms and mesial temporal lobe epilepsy with hippocampal sclerosis. Eur. J. Neurol..

[116] Lv R.-J., He J.-S., Fu Y.-H., Zhang Y.-Q., Shao X.-Q., Wu L.-W., Lu Q., Jin L.-R., Liu H. (2011). ASIC1a polymorphism is associated with temporal lobe epilepsy. Epilepsy Res..

[117] Pernhorst K., Raabe A., Niehusmann P., Van Loo K.M., Grote A., Hoffmann P., Cichon S., Sander T., Schoch S., Becker A.J. (2011). Promoter Variants Determine γ-Aminobutyric Acid Homeostasis-Related Gene Transcription in Human Epileptic Hippocampi. J. Neuropathol. Exp. Neurol..

[118] Heuser K., Nagelhus E.A., Taubøll E., Indahl U., Berg P.R., Lien S., Nakken S., Gjerstad L., Ottersen O.P. (2010). Variants of the genes encoding AQP4 and Kir4.1 are associated with subgroups of patients with temporal lobe epilepsy. Epilepsy Res..

[119] Shen N., Zhu X., Lin H., Li J., Li L., Niu F., Liu A., Wu X., Wang Y., Liu Y. (2015). Role of BDNF Val66Met functional polymorphism in temporal lobe epilepsy. Int. J. Neurosci..

[120] Alcantara J.A., Vincentiis S., Santos B., Kerr D., De Paula V., Alessi R., Linden H., Chaim T., Serpa M.H., Busatto G. (2018). BDNF Val66Met polymorphism is not related with temporal lobe epilepsy caused by hippocampal sclerosis in Brazilian population. Seizure.

[121] Lohoff F.W., Ferraro T.N., Dahl J.P., Hildebrandt M.A., Scattergood T.M., O’Connor M.J., Sperling M.R., Dlugos D.J., Berrettini W.H., Buono R.J. (2005). Lack of association between variations in the brain-derived neurotrophic factor (BDNF) gene and temporal lobe epilepsy. Epilepsy Res..

[122] Li X., Wang Y., Gu J., Meng Q., Gao Y., Zhao H., Yin Z. (2014). No association between polymorphisms in the calcium homeostasis modulator 1 gene and mesial temporal lobe epilepsy risk in a Chinese population. Seizure.

[123] Lv R.-J., He J.-S., Fu Y.-H., Shao X.-Q., Wu L.-W., Lu Q., Jin L.-R., Liu H. (2011). A polymorphism in CALHM1 is associated with temporal lobe epilepsy. Epilepsy Behav..

[124] Jamali S., Salzmann A., Perroud N., Ponsole-Lenfant M., Cillario J., Roll P., Roeckel-Trevisiol N., Crespel A., Balzar J., Schlachter K. (2010). Functional Variant in Complement C3 Gene Promoter and Genetic Susceptibility to Temporal Lobe Epilepsy and Febrile Seizures. PLoS ONE.

[125] Salzmann A., Guipponi M., Lyons P.J., Fricker L.D., Sapio M., Lambercy C., Buresi C., Bencheikh B.O.A., Lahjouji F., Ouazzani R. (2011). Carboxypeptidase A6 gene (CPA6) mutations in a recessive familial form of febrile seizures and temporal lobe epilepsy and in sporadic temporal lobe epilepsy. Hum. Mutat..

[126] Gambardella A., Manna I., Labate A., Chifari R., La Russa A., Serra P., Cittadella R., Bonavita S., Andreoli V., Lepiane E. (2003). GABA(B) receptor 1 polymorphism (G1465A) is associated with temporal lobe epilepsy. Neurology.

[127] Kauffman M., Levy E.M., Consalvo D., Mordoh J., Kochen S. (2008). GABABR1 (G1465A) gene variation and temporal lobe epilepsy controversy: New evidence. Seizure.

[128] Wang X., Sun W., Zhu X., Li L., Wu X., Lin H., Zhu S., Liu A., Du T., Liu Y. (2008). Association between the γ-aminobutyric acid type B receptor 1 and 2 gene polymorphisms and mesial temporal lobe epilepsy in a Han Chinese population. Epilepsy Res..

[129] Guipponi M., Chentouf A., Webling K.E., Freimann K., Crespel A., Nobile C., Lemke J.R., Hansen J., Dorn T., Lesca G. (2015). Galanin pathogenic mutations in temporal lobe epilepsy. Hum. Mol. Genet..

[130] Manna I., Labate A., Mumoli L., Palamara G., Ferlazzo E., Aguglia U., Quattrone A., Gambardella A. (2012). A Functional Genetic Variation of the 5-HTR2A Receptor Affects Age at Onset in Patients with Temporal Lobe Epilepsy. Ann. Hum. Genet..

[131] Li J., Lin H., Zhu X., Li L., Wang X., Sun W., Wu X., Liu A., Niu F., Wang Y. (2011). Association study of functional polymorphisms in serotonin transporter gene with temporal lobe epilepsy in Han Chinese population. Eur. J. Neurol..

[132] Schenkel L.C., Bragatti J.A., Becker J.A., Torres C.M., Martin K.C., De Souza A.C., Manfro G.G., Leistner-Segal S., Bianchin M.M. (2012). Serotonin gene polymorphisms and psychiatry comorbidities in temporal lobe epilepsy. Epilepsy Res..

[133] Manna I., Labate A., Gambardella A., Forabosco P., La Russa A., Le Piane E., Aguglia U., Quattrone A. (2007). Serotonin transporter gene (5-Htt): Association analysis with temporal lobe epilepsy. Neurosci. Lett..

[134] Salzmann A., Perroud N., Crespel A., Lambercy C., Malafosse A. (2008). Candidate genes for temporal lobe epilepsy: A replication study. Neurol. Sci..

[135] Liu Z., Yin X., Liu L., Tao H., Zhou H., Ma G., Cui L., Li Y., Zhang S., Xu Z. (2015). Association of KEAP1 and NFE2L2 polymorphisms with temporal lobe epilepsy and drug resistant epilepsy. Gene.

[136] Busolin G., Malacrida S., Bisulli F., Striano P., Di Bonaventura C., Egeo G., Pasini E., Cianci V., Ferlazzo E., Bianchi A. (2011). Association of intronic variants of the KCNAB1 gene with lateral temporal epilepsy. Epilepsy Res..

[137] Torres C.M., Siebert M., Bock H., Mota S.M., Krammer B.R., Duarte J.Á., Bragatti J.A., Castan J.U., De Castro L.A., Saraiva-Pereira M.L. (2017). NTRK2 (TrkB gene) variants and temporal lobe epilepsy: A genetic association study. Epilepsy Res..

[138] Zhu W.-Y., Jiang P., He X., Cao L.-J., Zhang L.-H., Dang R.-L., Tang M.-M., Xue Y., Li H.-D. (2015). Contribution of NRG1 Gene Polymorphisms in Temporal Lobe Epilepsy. J. Child Neurol..

[139] Stogmann E., Zimprich A., Baumgartner C., Aull-Watschinger S., Höllt V., Zimprich F. (2002). A functional polymorphism in the prodynorphin gene promotor is associated with temporal lobe epilepsy. Ann. Neurol..

[140] Bovo G., Diani E., Bisulli F., Di Bonaventura C., Striano P., Gambardella A., Ferlazzo E., Egeo G., Mecarelli O., Elia M. (2008). Analysis of LGI1 promoter sequence, PDYN and GABBR1 polymorphisms in sporadic and familial lateral temporal lobe epilepsy. Neurosci. Lett..

[141] Labate A., Manna I., Gambardella A., Le Piane E., La Russa A., Condino F., Cittadella R., Aguglia U., Quattrone A. (2007). Association between the M129V variant allele of PRNP gene and mild temporal lobe epilepsy in women. Neurosci. Lett..

[142] Manna I., Labate A., Mumoli L., Ferlazzo E., Aguglia U., Quattrone A., Gambardella A. (2013). No evidence for a role of the coding variant of the Toll-like receptor 4 gene in temporal lobe epilepsy. Seizure.

[143] Han W., Jiang P., Guo Y., Xu P., Dang R., Li G., He X., Liao D., Yan G. (2019). Role of t-PA and PAI-1 variants in temporal lobe epilepsy in Chinese Han population. BMC Neurol..

[144] Kasperavičiūtė D., Catarino C.B., Matarin M., Leu C., Novy J., Tostevin A., Leal B., Hessel E.V.S., Hallmann K., Hildebrand M.S. (2013). Epilepsy, hippocampal sclerosis and febrile seizures linked by common genetic variation around SCN1A. Brain.

[145] Suzuki T., Delgado-Escueta A.V., Aguan K., Alonso M.E., Shi J., Hara Y., Nishida M., Numata T., Medina M.T., Takeuchi T. (2004). Mutations in EFHC1 cause juvenile myoclonic epilepsy. Nat. Genet..

[146] Loucks C.M., Park K., Walker D.S., McEwan A.H., Timbers T.A., Ardiel E.L., Grundy L.J., Li C., Johnson J.-L., Kennedy J. (2019). EFHC1, implicated in juvenile myoclonic epilepsy, functions at the cilium and synapse to modulate dopamine signaling. eLife.

[147] De Nijs L., Lakaye B., Coumans B., Léon C., Ikeda T., Delgado-Escueta A.V., Grisar T., Chanas G. (2006). EFHC1, a protein mutated in juvenile myoclonic epilepsy, associates with the mitotic spindle through its N-terminus. Exp. Cell Res..

[148] Woermann F.G., Sisodiya S.M., Free S.L., Duncan J.S. (1998). Quantitative MRI in patients with idiopathic generalized epilepsy. Evidence of widespread cerebral structural changes. Brain.

[149] Library (2015). Juvenile Myoclonic Epilepsy: Genes.

[150] Saegusa H., Kurihara T., Zong S., Minowa O., Kazuno A.-A., Han W., Matsuda Y., Yamanaka H., Osanai M., Noda T. (2000). Altered pain responses in mice lacking alpha 1E subunit of the voltage-dependent Ca2+ channel. Proc. Natl. Acad. Sci. USA.

[151] Kanno T., Kanno Y., Siegel R.M., Jang M.K., Lenardo M.J., Ozato K. (2004). Selective Recognition of Acetylated Histones by Bromodomain Proteins Visualized in Living Cells. Mol. Cell.

[152] Crowley T., Brunori M., Rhee K., Wang X., Wolgemuth D.J. (2004). Change in nuclear-cytoplasmic localization of a double-bromodomain protein during proliferation and differentiation of mouse spinal cord and dorsal root ganglia. Dev. Brain Res..

[153] Gyuris A., Donovan D.J., Seymour K.A., Lovasco L.A., Smilowitz N.R., Halperin A.L.P., Klysik J.E., Freiman R.N. (2009). The chromatin-targeting protein Brd2 is required for neural tube closure and embryogenesis. Biochim. Biophys. Acta (Bba) Bioenerg..

[154] Velíšek L., Shang E., Velíšková J., Chachua T., Macchiarulo S., Maglakelidze G., Wolgemuth D.J., Greenberg D.A. (2011). GABAergic Neuron Deficit As An Idiopathic Generalized Epilepsy Mechanism: The Role Of BRD2 Haploinsufficiency In Juvenile Myoclonic Epilepsy. PLoS ONE.

[155] Pathak S., Miller J., Morris E.C., Stewart W.C.L., Greenberg D.A. (2018). DNA methylation of the BRD 2 promoter is associated with juvenile myoclonic epilepsy in Caucasians. Epilepsia.

[156] Schulz H., Ruppert A., Zara F., Madia F., Iacomino M., Vari M.S., Balagura G., Minetti C., Striano P., Bianchi A. (2019). No evidence for a BRD 2 promoter hypermethylation in blood leukocytes of Europeans with juvenile myoclonic epilepsy. Epilepsia.

[157] Cossette P., Liu L., Brisebois K., Dong H., Lortie A., Vanasse M., Saint-Hilaire J.-M., Carmant L., Verner A., Lu W.-Y. (2002). Mutation of GABRA1 in an autosomal dominant form of juvenile myoclonic epilepsy. Nat. Genet..

[158] Cossette P. (2010). Channelopathies and juvenile myoclonic epilepsy. Epilepsia.

[159] Gallagher M.J., Ding L., Maheshwari A., Macdonald R.L. (2007). The GABAA receptor 1 subunit epilepsy mutation A322D inhibits transmembrane helix formation and causes proteasomal degradation. Proc. Natl. Acad. Sci. USA.

[160] Ding L., Feng H., Macdonald R.L., Botzolakis E.J., Hu N., Gallagher M.J. (2010). GABAA receptor α1 subunit mutation A322D associated with autosomal dominant juvenile myoclonic epilepsy reduces the expression and alters the composition of wild type GABAA receptors. J. Biol..

[161] Macdonald R.L., Gallagher M.J., Feng H.-J., Kang J. (2004). GABAA receptor epilepsy mutations. Biochem. Pharm..

[162] Dibbens L.M., Feng H.-J., Richards M.C., Harkin L.A., Hodgson B.L., Scott D., Jenkins M., Petrou S., Sutherland G.R., Scheffer I.E. (2004). GABRD encoding a protein for extra or peri-synaptic GABAA receptors is a susceptibility locus for generalized epilepsies. Hum. Mol. Genet..

[163] Bhat M.A., Guru S.A., Mir R., Waza A.A., Zuberi M., Sumi M.P., Bodeliwala S., Puri V., Saxena A. (2018). Association of GABAA Receptor Gene with Epilepsy Syndromes. J. Mol. Neurosci..

[164] Lenzen K.P., Heils A., Lorenz S., Hempelmann A., Sander T. (2005). Association analysis of the Arg220His variation of the human gene encoding the GABA δ subunit with idiopathic generalized epilepsy. Epilepsy Res..

[165] Bando S.Y., Alegro M.C., Amaro E., Silva A.V., Castro L.H.M., Wen H.-T., Lima L.D.A., Brentani H.P., Moreira-Filho C.A. (2011). Hippocampal CA3 Transcriptome Signature Correlates with Initial Precipitating Injury in Refractory Mesial Temporal Lobe Epilepsy. PLoS ONE.

[166] Bymaster F.P., Carter P.A., Yamada M., Gomeza J., Wess J., Hamilton S.E., Nathanson N.M., McKinzie D.L., Felder C.C. (2003). Role of specific muscarinic receptor subtypes in cholinergic parasympathomimetic responses, in vivophosphoinositide hydrolysis, and pilocarpine-induced seizure activity. Eur. J. Neurosci..

[167] Vlaskamp D.R., Rump P., Callenbach P.M., Vos Y.J., Sikkema-Raddatz B., Van Ravenswaaij-Arts C.M., Brouwer O.F., Van Ravenswaaij C.M. (2016). Haploinsufficiency of the STX1B gene is associated with myoclonic astatic epilepsy. Eur. J. Paediatr. Neurol..

[168] Kang J.Q. (2017). Defects at the crossroads of GABAergic signaling in generalized genetic epilepsies. Epilepsy Res..

[169] Pearl P.L. (2018). Epilepsy syndromes in childhood. Continuum.

[170] Stogmann E., Lichtner P., Baumgartner C., Bonelli S., Assem-Hilger E., Leutmezer F., Schmied M., Hotzy C., Strom T.M., Meitinger T. (2006). Idiopathic generalized epilepsy phenotypes associated with different EFHC1 mutations. Neurology.

[171] Van Luijtelaar E., Budziszewska B., Jaworska-Feil L., Ellis J., Coenen A., Lasoń W. (2001). The ovarian hormones and absence epilepsy: A long-term EEG study and pharmacological effects in a genetic absence epilepsy model. Epilepsy Res..

[172] Jenz D. (2000). Epilepsy with grand mal on awakening and sleep-waking cycle. Clin. Neurophysiol..

[173] Lee C.G., Lee J., Lee M. (2018). Multi-gene panel testing in Korean patients with common genetic generalized epilepsy syndromes. PLoS ONE.

[174] Maurer-Morelli C.V., Secolin R., Morita M.E., Domingues R.R., Marchesini R.B., Santos N.F., Kobayashi E., Cendes F., Lopes-Cendes I. (2012). A Locus Identified on Chromosome18P11.31 is Associated with Hippocampal Abnormalities in a Family with Mesial Temporal Lobe Epilepsy. Front. Neurol..

[175] Catterall W.A. Sodium channel mutations and epilepsy. InJasper’s Basic Mechanisms of the Epilepsies 4th edition 2012. National Center for Biotechnology Information (US). National Center for Biotechnology Information 2012. https://www.ncbi.nlm.nih.gov/books/NBK98185/.

[176] Marban E., Yamagishi T., Tomaselli G.F. (1998). Structure and function of voltage-gated sodium channels. J. Physiol..

[177] Bhat M.A., Guru S.A., Mir R., Waza A.A., Zuberi M., Sumi M., Bodeliwala S., Samadhiya A., Puri V., Saxena A. (2018). Role of SCN1A and SCN2A Gene Polymorphisms in Epilepsy Syndromes-A Study from India. J. Neurol. Neurosci..

[178] Heinzen E.L., Yoon W., Tate S.K., Sen A., Wood N., Sisodiya S.M., Goldstein D.B. (2007). Nova2 Interacts with a Cis-Acting Polymorphism to Influence the Proportions of Drug-Responsive Splice Variants of SCN1A. Am. J. Hum. Genet..

[179] Dreses-Werringloer U., Vingtdeux V., Zhao H., Chandakkar P., Davies P., Marambaud P. (2013). CALHM1 controls the Ca2+-dependent MEK, ERK, RSK and MSK signaling cascade in neurons. J. Cell Sci..

[180] DeLorenzo R.J., Sun D.A., Deshpande L.S. (2006). Erratum to “Cellular mechanisms underlying acquired epilepsy: The calcium hypothesis of the induction and maintenance of epilepsy. Pharmacol. Ther..

[181] Dreses-Werringloer U., Lambert J.-C., Vingtdeux V., Zhao H., Vais H., Siebert A., Jain A., Koppel J., Rovelet-Lecrux A., Hannequin D. (2008). A Polymorphism in CALHM1 Influences Ca2+ Homeostasis, Aβ Levels, and Alzheimer’s Disease Risk. Cell.

[182] Palop J.J. (2009). Epilepsy and Cognitive Impairments in Alzheimer Disease. Arch. Neurol..

[183] Westmark C.J., Westmark P.R., Beard A.M., Hildebrandt S.M., Malter J.S. (2008). Seizure Susceptibility and Mortality in Mice that Over-Express Amyloid Precursor Protein. Int. J. Clin. Exp. Pathol..

[184] MacKenzie I.R., Miller L.A. (1994). Senile plaques in temporal lobe epilepsy. Acta Neuropathol..

[185] Raza M., Pal S., Rafiq A., DeLorenzo R.J. (2001). Long-term alteration of calcium homeostatic mechanisms in the pilocarpine model of temporal lobe epilepsy. Brain Res..

[186] Minkeviciene R., Rheims S., Dobszay M.B., Zilberter M., Hartikainen J., Fülöp L., Penke B., Zilberter Y., Harkany T., Pitkänen A. (2009). Amyloid β-Induced Neuronal Hyperexcitability Triggers Progressive Epilepsy. J. Neurosci..

[187] Billinton A., Baird V.H., Thom M., Duncan J.S., Upton N., Bowery N.G. (2001). GABAB(1) mRNA expression in hippocampal sclerosis associated with human temporal lobe epilepsy. Mol. Brain Res..

[188] Cavalleri G.L., Lynch J.M., Depondt C., Burley M.-W., Wood N.W., Sisodiya S.M., Goldstein D.B. (2005). Failure to replicate previously reported genetic associations with sporadic temporal lobe epilepsy: Where to from here?. Brain.

[189] Ma S., Abou-Khalil B., Sutcliffe J.S., Haines J.L., Hedera P. (2005). The GABBR1 locus and the G1465A variant is not associated with temporal lobe epilepsy preceded by febrile seizures. BMC Med Genet..

[190] Salzmann A., Moulard B., Crespel A., Buresi C., Malafosse A., Baldy-Moulinier M. (2005). GABAB Receptor 1 Polymorphism (G1465A) and Temporal Lobe Epilepsy. Epilepsia.

[191] Tan N.C.K., Heron S.E., Scheffer I.E., Berkovic S.F., Mulley J.C. (2005). Is Variation in the GABA(B) Receptor 1 Gene Associated with Temporal Lobe Epilepsy?. Epilepsia.

[192] Stogmann E., Zimprich A., Baumgartner C., Gleiss A., Zimprich F. (2006). Lack of Association between a GABAB Receptor 1 Gene Polymorphism and Temporal Lobe Epilepsy. Epilepsia.

[193] Ren L., Jin L., Zhang B., Jia Y., Wu L., Shen Y. (2005). Lack of GABABR1 gene variation (G1465A) in a Chinese population with temporal lobe epilepsy. Seizure.

[194] Nagelhus E.A., Mathiisen T., Ottersen O. (2004). Aquaporin-4 in the central nervous system: Cellular and subcellular distribution and coexpression with KIR4.1. Neuroscience.

[195] Amiry-Moghaddam M., Williamson A., Palomba M., Eid T., De Lanerolle N.C., Nagelhus E.A., Adams M.E., Froehner S.C., Agre P., Ottersen O.P. (2003). Delayed K+ clearance associated with aquaporin-4 mislocalization: Phenotypic defects in brains of -syntrophin-null mice. Proc. Natl. Acad. Sci. USA.

[196] Binder D.K., Yao X., Zador Z., Sick T.J., Verkman A.S., Manley G.T. (2006). Increased seizure duration and slowed potassium kinetics in mice lacking aquaporin-4 water channels. Glia.

[197] Lee D.J., Hsu M.S., Seldin M.M., Arellano J.L., Binder D.K. (2012). Decreased expression of the glial water channel aquaporin-4 in the intrahippocampal kainic acid model of epileptogenesis. Exp. Neurol..

[198] Binder D.K., Nagelhus E.A., Ottersen O.P. (2012). Aquaporin-4 and epilepsy. Glia.

[199] Eid T., Lee T.-S.W., Thomas M.J., Amiry-Moghaddam M., Bjørnsen L.P., Spencer D.D., Agre P., Ottersen O.P., De Lanerolle N.C. (2005). Loss of perivascular aquaporin 4 may underlie deficient water and K+ homeostasis in the human epileptogenic hippocampus. Proc. Natl. Acad. Sci. USA.

[200] Lesch K.-P., Mössner R. (1998). Genetically driven variation in serotonin uptake: Is there a link to affective spectrum, neurodevelopmental, and neurodegenerative disorders?. Biol. Psychiatry.

[201] Kauffman M., Consalvo D., González-Morón D., Aguirre F., D’Alessio L., Kochen S. (2009). Serotonin transporter gene variation and refractory mesial temporal epilepsy with hippocampal sclerosis. Epilepsy Res..

[202] Hrvoje H., Jasminka S., Lipa C.-S., Vida D., Branimir J. (2010). Association of serotonin transporter promoter (5-HTTLPR) and intron 2 (VNTR-2) polymorphisms with treatment response in temporal lobe epilepsy. Epilepsy Res..

[203] Schenkel L.C., Bragatti J.A., Torres C.M., Martin K.C., Manfro G.G., Leistner-Segal S., Bianchin M.M. (2011). Serotonin transporter gene (5HTT) polymorphisms and temporal lobe epilepsy. Epilepsy Res..

[204] Che F., Wei Y., Heng X., Fu Q., Jiang J. (2010). Association between serotonin transporter gene polymorphisms and non-lesional temporal lobe epilepsy in a Chinese Han population★. Neural Regen. Res..

[205] Stefulj J., Bordukalo-Niksic T., Hecimovic H., Demarin V., Jernej B. (2010). Epilepsy and serotonin (5HT): Variations of 5HT-related genes in temporal lobe epilepsy. Neurosci. Lett..

[206] Zimprich A., Kraus J., Wöltje M., Mayer P., Rauch E., Hollt V. (2000). An allelic variation in the human prodynorphin gene promoter alters stimulus-induced expression. J. Neurochem..

[207] Gambardella A., Manna I., Labate A., Chifari R., Serra P., La Russa A., Lepiane E., Cittadella R., Andreoli V., Sasanelli F. (2003). Prodynorphin gene promoter polymorphism and temporal lobe epilepsy. Epilepsia.

[208] Tilgen N., Rebstock J., Horvath S., Propping P., Elger C.E., Heils A. (2003). Prodynorphin gene promoter polymorphism and temporal lobe epilepsy. Ann. Neurol..

[209] Zhang N., Ouyang T.-H., Zhou Q., Kang H.-C., Zhu S. (2015). Prodynorphin gene promoter polymorphism and temporal lobe epilepsy: A meta-analysis. Acta Acad. Med. Wuhan.

[210] Waldmann R., Champigny G., Bassilana F., Heurteaux C., Lazdunski M. (1997). A proton-gated cation channel involved in acid-sensing. Nat. Cell Biol..

[211] Chen X., Kalbacher H., Gründer S. (2005). The Tarantula Toxin Psalmotoxin 1 Inhibits Acid-sensing Ion Channel (ASIC) 1a by Increasing Its Apparent H+ Affinity. J. Gen. Physiol..

[212] Wu H., Wang C., Liu B., Li H., Zhang Y., Dong S., Gao G., Zhang H. (2015). Altered Expression Pattern of Acid-Sensing Ion Channel Isoforms in Piriform Cortex After Seizures. Mol. Neurobiol..

[213] Yang F., Sun X., Ding Y., Ma H., Yang T.O., Ma Y., Wei D., Li W., Xu T., Jiang W. (2016). Astrocytic acid-sensing ion channel 1a contributes to the development of chronic epileptogenesis. Sci. Rep..

[214] Ziemann A.E., Schnizler M.K., Albert G.W., Severson M.A., Iii M.A.H., Welsh M.J., Wemmie J.A. (2008). Seizure termination by acidosis depends on ASIC1a. Nat. Neurosci..

[215] Gambardella A., Aguglia U., Cittadella R., Romeo N., Sibilia G., Lepiane E., Messina D., Manna I., Oliveri R.L., Zappia M. (1999). Apolipoprotein E polymorphisms and the risk of nonlesional temporal lobe epilepsy. Epilepsia.

[216] Gambardella A., Aguglia U., Chifari R., Labate A., Manna I., Serra P., Romeo N., Sibilia G., Lepiane E., La Russa A. (2005). ApoE Epsilon4 Allele and Disease Duration Affect Verbal Learning in Mild Temporal Lobe Epilepsy. Epilepsia.

[217] Kauffman M., Consalvo D., Moron D.G., Lereis V.P., Kochen S. (2010). ApoE ɛ4 genotype and the age at onset of temporal lobe epilepsy: A case–control study and meta-analysis. Epilepsy Res..

[218] Busch R.M., Lineweaver T.T., Naugle R.I., Kim K.H., Gong Y., Tilelli C.Q., Prayson R.A., Bingaman W., Najm I.M., Diaz-Arrastia R. (2007). ApoE- 4 is associated with reduced memory in long-standing intractable temporal lobe epilepsy. Neurol..

[219] Chapin J.S., Busch R., Janigro D., Dougherty M., Tilelli C.Q., Lineweaver T.T., Naugle R.I., Diaz-Arrastia R., Najm I.M. (2008). APOE ɛ4 is associated with postictal confusion in patients with medically refractory temporal lobe epilepsy. Epilepsy Res..

[220] Kauffman M., Pereira-De-Silva N., Consalvo D., Kochen S. (2009). ApoE ɛ4 is not associated with posictal confusion in patients with mesial temporal lobe epilepsy with hippocampal sclerosis. Epilepsy Res..

[221] Fu Y.-H., Lv R.-J., Jin L.-R., Lu Q., Shao X.-Q., He J.-S., Wu L.-W., Zhang L.-S., Hu H.-G. (2010). Association of apolipoprotein E polymorphisms with temporal lobe epilepsy in a Chinese Han population. Epilepsy Res..

[222] Li Z., Ding C., Gong X., Wang X., Cui T. (2016). Apolipoprotein E ε4 Allele was Associated with Nonlesional Mesial Temporal Lobe Epilepsy in Han Chinese Population. Medicine.

[223] Polvikoski T., Sulkava R., Haltia M., Kainulainen K., Vuorio A., Verkkoniemi A., Niinistö L., Halonen P., Kontula K. (1995). Apolipoprotein E, Dementia, and Cortical Deposition of β-Amyloid Protein. N. Engl. J. Med..

[224] Sheng J.G., Boop F.A., Mrak R.E., Griffin W.S.T. (2002). Increased Neuronal β-Amyloid Precursor Protein Expression in Human Temporal Lobe Epilepsy: Association with Interleukin-1α Immunoreactivity. J. Neurochem..

[225] Kodam A., Ourdev D., Maulik M., Hariharakrishnan J., Banerjee M., Wang Y., Kar S. (2018). A role for astrocyte-derived amyloid β peptides in the degeneration of neurons in an animal model of temporal lobe epilepsy. Brain Pathol..

[226] Nakagawara A., Liu X.-G., Ikegaki N., White P.S., Yamashiro D.J., Nycum L.M., Biegel J.A., Brodeur G.M. (1995). Cloning and chromosomal localization of the human TRK-B tyrosine kinase receptor gene (NTRK2). Genomics.

[227] Wirrell E.C., Grossardt B.R., So E.L., Nickels K.C. (2011). A population-based study of long-term outcomes of cryptogenic focal epilepsy in childhood: Cryptogenic epilepsy is probably not symptomatic epilepsy. Epilepsia.

[228] Harkin L.A., McMahon J.M., Iona X., Dibbens L., Pelekanos J.T., Zuberi S.M., Sadleir L.G., Andermann E., Gill D., Farrell K. (2007). The spectrum of SCN1A-related infantile epileptic encephalopathies. Brain.

[229] Zucca C., Redaelli F., Epifanio R., Zanotta N., Romeo A., Lodi M., Veggiotti P., Airoldi G., Panzeri C., Romaniello R. (2008). Cryptogenic Epileptic Syndromes Related to SCN1A. Arch. Neurol..

[230] Manolio T.A., Collins F.S., Cox N.J., Goldstein D.B., Hindorff L.A., Hunter D.J., McCarthy M.I., Ramos E.M., Cardon L.R., Chakravarti A. (2009). Finding the missing heritability of complex diseases. Nat. Cell Biol..

[231] Allen A.S., Bellows S.T., Berkovic S.F., Bridgers J., Burgess R., Cavalleri G., Chung S.-K., Cossette P., Delanty N., Dlugos D. (2017). Ultra-rare genetic variation in common epilepsies: A case-control sequencing study. Lancet Neurol..

[232] Bennett C.A., Petrovski S., Oliver K.L., Berkovic S.F. (2017). ExACtly zero or once: A clinically helpful guide to assessing genetic variants in mild epilepsies. Neurol. Genet..

[233] May P., Girard A., Harrer M., Bobbili D.R., Schubert J., Wolking S., Becker F., Lachance-Touchette P., Meloche C., Gravel M. (2018). Rare coding variants in genes encoding GABAA receptors in genetic generalised epilepsies: An exome-based case-control study. Lancet Neurol..

[234] Feng Y.-C.A., Howrigan D.P., Abbott L.E., Tashman K., Cerrato F., Singh T., Heyne H., Byrnes A., Churchhouse C., Watts N. (2019). Ultra-Rare Genetic Variation in the Epilepsies: A Whole-Exome Sequencing Study of 17,606 Individuals. Am. J. Hum. Genet..

[235] Hindorff L.A., Sethupathy P., Junkins H.A., Ramos E.M., Mehta J.P., Collins F.S., Manolio T.A. (2009). Potential etiologic and functional implications of genome-wide association loci for human diseases and traits. Proc. Natl. Acad. Sci. USA.

[236] Leu C., Stevelink R., Smith A.W., Goleva S.B., Kanai M., Ferguson L., Campbell C., Kamatani Y., Okada Y., Sisodiya S.M. (2019). Polygenic burden in focal and generalized epilepsies. Brain.

[237] Speed D., O’Brien T.J., Palotie A., Shkura K., Marson A.G., Balding D.J., Johnson M.R. (2014). Describing the genetic architecture of epilepsy through heritability analysis. Brain.

[238] Ellis C.A., Petrovski S., Berkovic S.F. (2020). Epilepsy genetics: Clinical impacts and biological insights. Lancet Neurol..

[239] Martin A.R., Gignoux C.R., Walters R.K., Wojcik G.L., Neale B.M., Gravel S., Daly M.J., Bustamante C.D., Kenny E.E. (2017). Human Demographic History Impacts Genetic Risk Prediction across Diverse Populations. Am. J. Hum. Genet..

[240] Poduri A., Sheidley B.R., Shostak S., Ottman R. (2014). Genetic testing in the epilepsies—developments and dilemmas. Nat. Rev. Neurol..

[241] Kearney H., Byrne S., Cavalleri G.L., Delanty N. (2019). Tackling Epilepsy with High-definition Precision Medicine. JAMA Neurol..

[242] Carabotti M., Scirocco A., Maselli M.A., Severi C. (2015). The gut-brain axis: Interactions between enteric microbiota, central and enteric nervous systems. Ann. Gastroenterol. Q. Publ. Hell. Soc. Gastroenterol..

[243] Sharp A.J., Mefford H.C., Li K., Baker C., Skinner C., Stevenson R.E., Schroer R.J., Novara F., De Gregori M., Ciccone R. (2008). A recurrent 15q13.3 microdeletion syndrome associated with mental retardation and seizures. Nat. Genet..

[244] Helbig I., Mefford H.C., Sharp A.J., Guipponi M., Fichera M., Franke A., Muhle H., De Kovel C., Baker C., Von Spiczak S. (2009). 15q13.3 microdeletions increase risk of idiopathic generalized epilepsy. Nat. Genet..

[245] Dibbens L.M., Mullen S., Helbig I., Mefford H.C., Bayly M.A., Bellows S., Leu C., Trucks H., Obermeier T., Wittig M. (2009). Familial and sporadic 15q13.3 microdeletions in idiopathic generalized epilepsy: Precedent for disorders with complex inheritance. Hum. Mol. Genet..

[246] De Kovel C.G.F., Trucks H., Helbig I., Mefford H.C., Baker C., Leu C., Kluck C., Muhle H., Von Spiczak S., Ostertag P. (2009). Recurrent microdeletions at 15q11.2 and 16p13.11 predispose to idiopathic generalized epilepsies. Brain.

[247] Mefford H.C., Yendle S.C., Hsu C., Cook J., Ba E.G., Bsc J.M.M., Eeg-Olofsson O., Sadleir L.G., Gill D., Ben-Zeev B. (2011). Rare copy number variants are an important cause of epileptic encephalopathies. Ann. Neurol..

[248] Møller R.S., Weber Y.G., Klitten L.L., Trucks H., Muhle H., Kunz W.S., Mefford H.C., Franke A., Kautza M., Wolf P. (2013). Exon-disrupting deletions ofNRXN1in idiopathic generalized epilepsy. Epilepsia.

[249] Lal D., Trucks H., Møller R.S., Hjalgrim H., Koeleman B.P., De Kovel C.G., Visscher F., Weber Y.G., Lerche H., Becker F. (2013). Rare exonic deletions of theRBFOX1gene increase risk of idiopathic generalized epilepsy. Epilepsia.

[250] Lionel A.C., Vaags A.K., Sato D., Gazzellone M.J., Mitchell E.B., Chen H.Y., Costain G., Walker S., Egger G., Thiruvahindrapuram B. (2013). Rare exonic deletions implicate the synaptic organizer Gephyrin (GPHN) in risk for autism, schizophrenia and seizures. Hum. Mol. Genet..

[251] Harrison V., Connell L., Hayesmoore J., McParland J., Pike M.G., Blair E. (2011). Compound heterozygous deletion of NRXN1 causing severe developmental delay with early onset epilepsy in two sisters. Am. J. Med Genet. Part A.

[252] Krepischi A.C.V., Knijnenburg J., Bertola D.R., Kim C.A., Pearson P.L., Bijlsma E., Szuhai K., Kok F., Vianna-Morgante A.M., Rosenberg C. (2010). Two distinct regions in 2q24.2-q24.3 associated with idiopathic epilepsy. Epilepsia.

[253] Naseer M.I., Faheem M., Chaudhary A.G., Kumosani T.A., AlQuaiti M., Jan M.M., Jamal H.S., Al-Qahtani M. (2015). Genome wide analysis of novel copy number variations duplications/deletions of different epileptic patients in Saudi Arabia. Bmc Genom..

[254] Mefford H.C., Muhle H., Ostertag P., Von Spiczak S., Buysse K., Baker C., Franke A., Malafosse A., Genton P., Thomas P. (2010). Genome-Wide Copy Number Variation in Epilepsy: Novel Susceptibility Loci in Idiopathic Generalized and Focal Epilepsies. PLoS Genet..

[255] Liu J.Y.W., Kasperaviciute D., Martinian L., Thom M., Sisodiya S. (2012). Neuropathology of 16p13.11 Deletion in Epilepsy. PLoS ONE.

[256] Dimassi S., Labalme A., Lesca G., Rudolf G., Bruneau N., Hirsch E., Arzimanoglou A., Motte J., De Saint-Martin A., Boutry-Kryza N. (2013). A subset of genomic alterations detected in rolandic epilepsies contains candidate or known epilepsy genes includingGRIN2AandPRRT2. Epilepsia.

[257] Strehlow V., Swinkels M.E., Thomas R.H., Rapps N., Syrbe S., Dorn T., Lemke J.R. (2016). Generalized Epilepsy and Myoclonic Seizures in 22q11.2 Deletion Syndrome. Mol. Syndr..

[258] Biervert C., Schroeder B.C., Kubisch C., Berkovic S.F., Propping P., Jentsch T.J., Steinlein O.K. (1998). A Potassium Channel Mutation in Neonatal Human Epilepsy. Science.

[259] Singh N.A., Charlier C., Stauffer D., Dupont B.R., Leach R.J., Melis R., Ronen G.M., Bjerre I., Quattlebaum T., Murphy J.V. (1998). A novel potassium channel gene, KCNQ2, is mutated in an inherited epilepsy of newborns. Nat. Genet..

[260] Maljevic S., Wuttke T., Seebohm G., Lerche H. (2010). K V 7 channelopathies. Pflügers Archiv-Eur. J. Physiol..

[261] Heron S.E., Crossland K.M., Andermann E., Phillips H.A., Hall A.J., Bleasel A., Shevell M., Mercho S., Seni M.-H., Guiot M.-C. (2002). Sodium-channel defects in benign familial neonatal-infantile seizures. Lancet.

[262] Scalmani P., Rusconi R., Armatura E., Zara F., Avanzini G., Franceschetti S., Mantegazza M. (2006). Effects in Neocortical Neurons of Mutations of the Nav1.2 Na+ Channel causing Benign Familial Neonatal-Infantile Seizures. J. Neurosci..

[263] Liao Y., Deprez L., Maljevic S., Pitsch J., Claes L., Hristova D., Jordanova A., Ala-Mello S., Bellan-Koch A., Blazevic D. (2010). Molecular correlates of age-dependent seizures in an inherited neonatal-infantile epilepsy. Brain.

[264] Berkovic S.F. (2015). Genetics of Epilepsy in Clinical Practice: Genetics of Epilepsy in Clinical Practice. Epilepsy Curr..

[265] Lü J.-J., Zhang Y., Chen Y.-C., Pan H., Wang J.-L., Zhang L., Wu H.-S., Xu K.-M., Liu X.-Y., Tao L.-D. (2005). T-type calcium channel gene-CACNA1H is a susceptibility gene to childhood absence epilepsy. Zhonghua Chin. J. Pediatr..

[266] Koeleman B.P., De Kovel C.G., Trenité D.G.K.-N. (2013). Photoparoxysmal EEG response and genetic dissection of juvenile myoclonic epilepsy. Epilepsy Behav..

[267] Caciagli L., Wandschneider B., Ecenteno M., Vollmar C., Vos S.B., Trimmel K., Long L., Xiao F., Lowe A.J., Sidhu M.K. (2020). Motor hyperactivation during cognitive tasks: An endophenotype of juvenile myoclonic epilepsy. Epilepsia.

[268] Alhusaini S., Whelan C.D., Dohertya C.P., Delanty N., Fitzsimons M., Cavalleri G.L. (2015). Temporal Cortex Morphology in Mesial Temporal Lobe Epilepsy Patients and Their Asymptomatic Siblings. Cereb. Cortex.

[269] Abbasi B., Goldenholz D.M. (2019). Machine learning applications in epilepsy. Epilepsia.

[270] Zhang J., Cheng W., Wang Z., Zhang Z., Lu W., Lu G.-M., Feng J. (2012). Pattern Classification of Large-Scale Functional Brain Networks: Identification of Informative Neuroimaging Markers for Epilepsy. PLoS ONE.

[271] Stewart J.D., Horvath R., Baruffini E., Ferrero I., Bulst S., Watkins P.B., Fontana R.J., Day C.P., Chinnery P.F. (2010). Polymerase γ gene POLG determines the risk of sodium valproate-induced liver toxicity. Hepatology.

[272] Ihtisham K., Ramanujam B., Srivastava S., Mehra N.K., Kaur G., Khanna N., Jain S., Kumar S., Kaul B., Samudrala R. (2019). Association of cutaneous adverse drug reactions due to antiepileptic drugs with HLA alleles in a North Indian population. Seizure.

[273] McCormack M., Alfirevic A., Bourgeois S., Farrell J.J., Kasperavičiūtė D., Carrington M., Sills G.J., Marson T., Jia X., de Bakker P.I. (2011). HLA-A*3101 and carbamazepine-induced hypersensitivity reactions in Europeans. N. Engl. J. Med..

[274] Registry N.-G. (2014). Carbamazepine Response. NCBI Genetic Testing Registry. https://www.ncbi.nlm.nih.gov/gtr/conditions/CN077964/.

[275] Silvado C.E., Terra V.C., Twardowschy C.A. (2018). CYP2C9 polymorphisms in epilepsy: Influence on phenytoin treatment. Pharm. Pers. Med..

[276] Chaudhary N., Kabra M., Gulati S., Gupta Y.K., Pandey R.M., Bhatia B.D. (2016). Frequencies of CYP2C9 polymorphisms in North Indian population and their association with drug levels in children on phenytoin monotherapy. BMC Pediatr..

[277] Caudle K.E., Rettie A.E., Whirl-Carrillo M., Smith L.H., Mintzer S., Lee M.T.M., Klein T.E., Callaghan J.T. (2014). Clinical Pharmacogenetics Implementation Consortium Guidelines for CYP2C9 and HLA-B Genotypes and Phenytoin Dosing. Clin. Pharm..

[278] Krishnamoorthy E.S. (2001). Psychiatric issues in epilepsy. Curr Opin Neurol..

