# Supplementary material for: Genetic Landscape of Common Epilepsies: Advancing towards Precision in Treatment
Source: Int J Mol Sci. 2020 Oct 21;21(20):7784. doi: 10.3390/ijms21207784 (PMC7589654; doi:10.3390/ijms21207784)
Supplement: Supplementary file 1 [file ijms-21-07784-s001.pdf]

Supplementary data

Table S1: List of gene panels used for epilepsy diagnosis in different companies.

Sl. No.	Company Name (Country of origin)	Phenotype Diagnosed	Gene Panel
1	Athena Diagnostics Inc (US)	Epilepsy	*ALDH7A1, CACNA1A, CASR, CHRNA2, CHRNA4, CHRNB2, CSTB, DEPDC5, EFHC1,EPM2A, GABRA1, GABRB3, GABRD, GABRG2, GRIN2A, KCNMA1, KCNQ2, KCNQ3,KCNT1, KCTD7, LGI1, MBD5, NHLRC1, PCDH19, PRICKLE1, PRICKLE2, PRRT2,SCARB2, SCN1A, SCN1B, SCN2A, SCN9A, SLC2A1, SLC4A10, TBC1D24, ABAT, ADSL,ALG13, ALG9, AMT, ASAH1, ASPM, ATP1A3 , BCKDK, BRAT1, CACNA1H, CACNB4,CHD2, CHRNA7 , CPA6, CRH, CYP27A1, DYNC1H1, FOLR1, GABRB2 , GAMT, GATM, GLDC, GOSR2, GRIN2B, HCN1, HCN4, KCNC1, KCNH2, L2HGDH, LIAS, LMNB2, NDUFA1, PHGDH, PIGO , PNPO, PRIMA1, SCN3A, SCN5A, SLC19A3, SLC25A19,SLC35A2, SLC6A1, SLC6A8, ST3GAL5, STX1B, SUCLA2, SYNJ1, ALPL, ARFGEF2, ARHGEF9, ATP2A2, ATP6AP2, ATP6V0A2, ATRX, CASK,CHRNA4, CLN3, CLN5, CLN6, CLN8, CPT2, CTSD, CUL4B, DCX,DNAJC5, EMX2, FGD1, FGFR3, FLNA, GPC3, GPR56, GRIA3,LAMA2, LARGE1, LBR, MFSD8, NIPBL, NRXN1, OFD1, OPHN1, PAFAH1B1, PAK3,PANK2, PAX6 , PEX7, PHF6, PIGV, PLA2G6, PNKP, PPT1, PQBP1,RAB39B, RAB3GAP1, RAI1, RELN, RNASEH2A, RNASEH2B, RNASEH2C, SAMHD1, SCARB2,SCN2A, SCN8A, SERPINI1, SETBP1, SLC25A22, SLC9A6, SMC1A, SMC3,SMS, SPTAN1, SRPX2, STXBP1, SYP, TBX1, TCF4, TPP1, TREX1, TUBA1A, TUBA8, TUBB2B, UBE3A, VPS13A, ZEB2, ARX, ATP1A2, CACNA1A, COL4A1,NOTCH3,CASR,CDKL5,VPS13B,CNTNAP2,CSTB, FKTN, FKRP, FOXG1, MECP2, MEF2C, PLP1, POLG, POMT1, POMT2, POMGNT1,SYNGAP1, TSC1, TSC2, WDR62, GFAP, MCPH1, ADGRV1, ALG13,ANKRD11, ATP13A2, ATP1A3, CACNA2D1, CACNA2D2, CENPJ, CHD2, CHRNA7, CTSF, DEAF1, DNM1, DOCK7, DPYD, DYRK1A, EEF1A2, GABRB2, GLDC, GNAO1, GOSR2, GRIN1, GRIN2B, GRN, HCN1, HCN4,HNRNPU, HPRT1, IQSEC2, KANSL1, KCNA2, KCNB1, KCNC1, KCNH2, KCNJ11, KIAA2022, L2HGDH, LIAS,LMNB2, MAGI2, NDE1, NDUFA1, NR2F1, PIGA, PIGN, PIGO, PLCB1, PURA,QARS, RBFOX1, ROGDI, SETD2, SHH, SIK1, SIX3, SLC13A5, SNAP25, SPATA5, ST3GAL3, STIL, SYN1,SZT2, TBL1XR1, TSEN54, WDR45*
2	Blueprint Genetics (Finland)	Juvenile myoclonic epilepsy (JME)	*GABRA1, EFHC1*
		Childhood absence epilepsy	*CACNA1H, GABRB3, GABRG2*
		Familial temporal lobe epilepsy	*LGI1, RELN, GAL, CPA6, MICAL1*
		Genetic generalized epilepsy	*SLC2A1, CACNB4*
		Epilepsy	*CLCN2, EFHC1, GABRA1, GABRB3, GABRG2, GRIN2A, KCNC1, RELN*
3	Emory Genetics Laboratory (US)	Epilepsy	*ABAT CENPJ DNAJC5 GRIN2A MBD5 PLCB1 SCN8A STXBP1 ADSL CHRNA2 EFHC1 HCN1 MCPH1 PNKP SCN9A SYN1 ALDH7A1 CHRNA4 EMX2 HCN4 MECP2 PNPO SHH TBC1D24 ARHGEF9 CHRNB2 EPM2A KCNA1 MEF2C POLG SIX3 TCF4 ARX CLN3 FLNA KCNJ10 MFSD8 PPT1 SLC19A3 TPP1*

			ASPM CLN5 FOLR1 KCNJ11 MTHFR PRICKLE1 SLC25A19 TSC1 ATP1A2 CLN6 FOXG1 KCNMA1 NDE1 PRICKLE2 SLC25A22 TSC2 ATP6AP2 CLN8 GABRA1 KCNQ2 NDUFA1 PRRT2 SLC2A1 TSEN54 BCKDK CNTNAP2 GABRG2 KCNQ3 NHLRC1 RELN SLC9A6 UBE3A CACNA1A CPA6 GAMT KCNT1 NRXN1 SCARB2 SPTAN1 WDR62 CACNB4 CSTB GATM KCTD7 OPHN1 SCN1A SRPX2 ZEB2 CASK CTSD GOSR2 LGI1 PAFAH1B1 SCN1B ST3GAL3 CASR CYP27A1 GPR56 LIAS PCDH19 SCN2A ST3GAL5 CDKL5 DCX GPR98 MAGI2 PHF6 SCN3A STIL
4	Eurofin Clinical Genetics (India)	Epilepsy	*ABAT, ADGRG1, ADGRV1, ADSL, ALDH5A1, ALDH7A1, ARHGEF9, ARX, ASPM, ATP1A2, ATP6AP2, BCKDK, CACNA1A, CACNB4, CASK, CASR, CDKL5, CENPJ, CHRNA2, CHRNA4, CHRNB2, CLN3, CLN5, CLN6, CLN8, CNTNAP2, CPA6, CSTB, CTSD, CYP27A1, DCX, DNAJC5, EFHC1, EMX2, EPM2A, FLNA, FOLR1, FOXG1, GABRA1, GABRG2, GAMT, GATM, GOSR2, GRIN2A, HCN1, HCN4, KCNA1, KCNJ10, KCNJ11, KCNMA1, KCNQ2, KCNQ3, KCNT1, KCTD7, LGI1, LIAS, MAGI2, MBD5, MCPH1, MECP2, MEF2C, MFSD8, MTHFR, NDE1, NDUFA1, NHLRC1, NRXN1, OPHN1, PAFAH1B1, PCDH19, PHF6, PLCB1, PNKP, PNPO, POLG, PPT1, PRICKLE1, PRICKLE2, PRRT2, RELN, SCARB2, SCN1A, SCN1B, SCN2A, SCN3A, SCN8A, SCN9A, SHH, SIX3, SLC19A3, SLC25A19, SLC25A22, SLC2A1, SLC9A6, SPTAN1, SRPX2, ST3GAL3, ST3GAL5, STIL, STXBP1, SYN1, TBC1D24, TCF4, TPP1, TSC1, TSC2, TSEN54, UBE3A, WDR62, ZEB2*
5	Gene Dx (USA)	Juvenile myoclonic epilepsy (JME)	*CACNB4, EFHC1, GABRA1, CILK*
6	Invitae (USA)	Epilepsy	*ADSL, ALDH5A1, ALDH7A1, ALG13, ARG1, ARHGEF9, ARX, ATP1A2, ATP1A3, ATRX, BRAT1, C12orf57, CACNA1A, CACNA2D2, CARS2, CASK, CDKL5, CHD2, CHRNA2, CHRNA4, CHRNB2, CLCN4, CLN2, (TPP1), CLN3, CLN5, CLN6, CLN8, CNTNAP2, CSTB, CTSD, DDC, DEPDC5, DNAJC5, DNM1, DOCK7, DYRK1A, EEF1A2, EFHC1, EHMT1, EPM2A, FARS2, FOLR1, FOXG1, FRRS1L, GABBR2, GABRA1, GABRB2, GABRB3, GABRG2, GAMT, GATM, GLRA1, GNAO1, GOSR2, GRIN1, GRIN2A, GRIN2B, HCN1, HNRNPU, IER3IP1, IQSEC2, ITPA, JMJD1C, KANSL1, KCNA2, KCNB1, KCNC1, KCNH2, KCNJ10, KCNMA1, KCNQ2, KCNQ3, KCNT1, KCTD7, LGI1, LIAS, MBD5, MECP2, MEF2C, MFSD8, MOCS1, MOCS2, MTOR, NEDD4L, NEXMIF, NGLY1, NHLRC1, NPRL3, NRXN1, PACS1, PCDH19, PIGA, PIGN, PIGO, PLCB1, PNKD, PNKP, PNPO, POLG, PPT1, PRICKLE1, PRIMA1, PRRT2, PURA, QARS, RELN, ROGDI, SATB2, SCARB2, SCN1A, SCN1B, SCN2A, SCN3A, SCN8A, SCN9A, SERPINI1, SGCE, SIK1, SLC12A5, SLC13A5, SLC19A3, SLC25A12, SLC25A22, SLC2A1, SLC35A2, SLC6A1, SLC6A8, SLC9A6, SMC1A, SNX27, SPATA5, SPTAN1, ST3GAL5, STRADA, STX1, B, STXBP1, SUOX, SYN1, SYNGAP1, SYNJ1, SZT2, TBC1D24, TCF4, TPK1, TSC1, TSC2, UBE3A, WDR45, WWOX, ZDHHC9, ZEB2, ABAT, ARHGEF15, ATP6AP2, CACNA1H, CACNB4, CASR, CERS1, CNTN2, CPA6, DIAPH1, FASN, GABRD, GAL, GPHN, KCNA1, KCND2, KCNH5, KPNA7, LMNB2, NECAP1, PIGG, PIGQ, PIK3AP1, PRDM8, PRICKLE2, RBFOX1, RBFOX3, RYR3, SCN5A, SETD2, SLC35A3, SNAP25, SRPX2, ST3GAL3, TBL1XR, AMT, GCSH, GLDC*

Table S2: List of available genes used for genetic diagnosis of rare epilepsies.

Sr. No.	Gene	Phenotype
1	*ALDH7A1*	Pyridoxine-dependent epilepsy
2	*BRAT1*	EE
3	*DEPDC5*	Tuberous sclerosis complex, focal epilepsy
4	*DNM1*	EIEE

5	*DOLK*	Congenital disorder of glycosylation, type Im
6	*FOLR1*	Folinic acid-responsive seizures
7	*FOXG1*	West syndrome
8	*GABRG2*	Childhood absence epilepsy, GEFS+, FS, EIEE
9	*GAMT*	Creatine deficiency syndrome 2
10	*GATM*	Creatine deficiency syndrome 3
11	*GNAO1*	EIEE
12	*GRIN2A*	Focal epilepsy and speech disorder with/without mental retardation
13	*GRIN2D*	EIEE
14	*HCN1*	EIEE, GEFS+
15	*KCNMA1*	Paroxysmal non-kinesigenic dyskinesia, with or without generalized epilepsy, idiopathic generalized epilepsy
16	*KCNQ2*	EIEE, benign familial neonatal seizures
17	*KCNQ3*	EIEE, benign familial neonatal seizures
18	*KCNT1*	Autosomal dominant nocturnal frontal lobe epilepsy, epilepsy of infancy with migrating focal seizures
19	*MEF2C*	West syndrome
20	*PCDH19*	Dravet syndrome-like EIEE/Juberg–Hellman syndrome
21	*PNPO*	Pyridoxal 5′-phosphate responsive epilepsy
22	*SCN1A*	Dravet syndrome; GEFS+
23	*SCN2A*	EIEE, infantile epilepsy with migrating focal seizures, West syndrome, Ohtahara syndrome, BFINS
24	*SCN8A*	EIEE
25	*SLC2A1*	Glut-1 deficiency
26	*SLC35A2*	Congenital disorder of glycosylation, type IIm
27	*AMACR*	Alpha-methylacyl-CoA racemase deficiency, bile acid synthesis defect
28	*CACNB4*	Episodic ataxia, epilepsy, idiopathic generalized, susceptibility to, 9
29	*CASR*	Hypocalcemia, neonatal hyperparathyroidism, familial hypocalciuric hypercalcemia with transient neonatal hyperparathyroidism
30	*CHRNA2*	Epilepsy, nocturnal frontal lobe
31	*CHRNA4*	Epilepsy, nocturnal frontal lobe
32	*CHRNB2*	Epilepsy, nocturnal frontal lobe
33	*CLCN2*	Leukoencephalopathy with ataxia, epilepsy
34	*EFHC1*	Epilepsy, myoclonic juvenile, epilepsy, severe intractable, epilepsy, juvenile absence
35	*GABRA1*	Epileptic encephalopathy, early infantile, epilepsy, childhood absence, epilepsy, juvenile myoclonic
36	*GRIN2A*	Epilepsy, focal, with speech disorder
37	*KCNA1*	Episodic ataxia/myokymia syndrome
38	*KCNC1*	Epilepsy, progressive myoclonic
39	*KCNQ2*	Epileptic encephalopathy, early infantile, benign familial neonatal seizures, myokymia
40	*MTOR*	Smith–Kingsmore syndrome
41	*NPRL3*	Epilepsy, familial focal, with variable foci 3
42	*POLG*	POLG-related ataxia neuropathy spectrum disorders, Sensory ataxia, dysarthria, and ophthalmoparesis, Alpers syndrome, progressive external ophthalmoplegia with mitochondrial DNA deletions, mitochondrial DNA depletion syndrome
43	*PRRT2*	Episodic kinesigenic dyskinesia, seizures, benign familial infantile, 2, convulsions, familial infantile, with paroxysmal choreoathetosis
44	*RELN*	Lissencephaly, epilepsy, familial temporal lobe
45	*SCN1B*	Atrial fibrillation, Brugada syndrome, generalized epilepsy with febrile seizures plus, epilepsy, generalized, with febrile seizures plus, type 1, epileptic encephalopathy, early infantile, 52
46	*SCN9A*	Paroxysmal extreme pain disorder, small fiber neuropathy, Erythermalgia, primary, generalized epilepsy with febrile seizures plus, type 7, insensitivity to pain, congenital, autosomal recessive
47	*SLC6A1*	Myoclonic-astastic epilepsy

48	*TBC1D24*	Deafness, onychodystrophy, osteodystrophy, mental retardation and seizures (DOOR) syndrome, deafness, autosomal dominant, 65, Myoclonic epilepsy, infantile, familial, epileptic encephalopathy, early infantile, 16, deafness, autosomal recessive 86

EE, Epileptic encephalopathy; EIEE, Early infantile epileptic encephalopathy; GEFS+, Generalized epilepsy with febrile seizure plus; FS, Febrile seizures; BFINS, Benign neonatal–infantile seizures; DNA, Deoxyribonucleic acid; DOOR, Deafness, onychodystrophy, osteodystrophy and mental retardation.
